# A Biologically Informed Vision‐Guided Framework for Interpretable T Cell Receptor–Epitope Binding Prediction

**DOI:** 10.1002/advs.202512544

**Published:** 2025-11-07

**Authors:** Yajing Yuan, Junwei Chen, Yufang Zhang, Yitian Fang, Zhongcheng Fang, Yanyi Chu, Jiayi Li, Chen Zhang, Yuzhe Li, Dongqing Wei

**Affiliations:** ^1^ State Key Laboratory of Microbial Metabolism Joint International Research Laboratory of Metabolic & Developmental Sciences and School of Life Sciences and Biotechnology Shanghai Jiao Tong University Shanghai 200040 P. R. China; ^2^ School of Mathematical Sciences and SJTU‐Yale Joint Center for Biostatistics and Data Science Shanghai Jiao Tong University Shanghai 200240 P. R. China; ^3^ Arc Institute, Palo Alto CA 94304 USA; ^4^ Zhongjing Research and Industrialization Institute of Chinese Medicine Zhongguancun Scientific Park, Meixi Nanyang Henan 473006 P. R. China; ^5^ Hebi Branch Henan Academy of Sciences Qishui Guang East, Qibin District Hebi Henan 458030 P. R. China; ^6^ Qihe Laboratory Qishui Guang East, Qibin District Hebi Henan 458030 P. R. China

**Keywords:** biologically informed modeling, cancer immunotherapy, physicochemical properties, TCR–epitope interaction, vision‐guided learning

## Abstract

Accurate identification of the interactions between T‐cell receptors (TCRs) and antigenic epitopes presented by major histocompatibility complex (MHC) molecules is fundamental to advancing cancer immunotherapy. Nevertheless, predictive modeling of TCR–epitope binding remains challenging, as existing models struggle to generalize to unseen epitopes while often overlooking key physicochemical properties governing immune recognition. Here, a biologically informed vision‐guided deep learning framework (DAISY) is proposed for robust and interpretable TCR–epitope binding prediction. DAISY integrates hierarchical physicochemical features via a biologically inspired Condition‐Adaptive Fusion module, jointly modeling residue‐level spatial interactions and global biochemical context. DAISY consistently outperforms state‐of‐the‐art models across four generalization scenarios, notably improving ROC‐AUC by 11% and PR‐AUC by 16% over the strongest competitor in the most challenging Unseen‐Pair setting. DAISY also offers intuitive interpretability by localizing interaction‐relevant residues via Score‐CAM visualizations. Furthermore, its computational predictions are bridged to key immunological and clinical outcomes, demonstrating utility in correlating with T‐cell clonal expansion, identifying functional TCRs, and robustly forecasting patient survival. Together, DAISY can serve as a powerful tool for broad translational immunology and introduces a scalable modeling paradigm for next‐generation immune modeling.

## Graphical abstract

Integrating hierarchical physicochemical features via a bio‐inspired design, the DAISY framework robustly predicts T‐cell receptor binding, significantly outperforming state‐of‐the‐art models on unseen epitopes. It uniquely offers visual interpretability via Score‐CAM visualizations and profound clinical relevance, correlating with T‐cell expansion, enabling functional TCR screening, and defining functional biomarkers that forecast patient survival in immunotherapy.

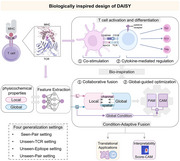

## 1 Introduction

T cells play a crucial role in regulating effector immune cells during the adaptive immune response to various pathogenic entities, including bacteria, viruses, and tumor cells.^[^ ^1^, ^2^ ^]^ Antigenic epitopes, presented by major histocompatibility complex (MHC) molecules, are recognized by T cell receptors (TCRs) on the surface of T cells, thereby eliminating infected or malignant cells and initiating appropriate immune responses.^[^ ^3^, ^4^ ^]^ Consequently, understanding the binding mechanisms underlying TCR–epitope interactions is essential for advancing personalized cancer immunotherapy, developing innovative vaccines,^[^ ^5^, ^6^ ^]^ and discovering antigens associated with autoimmune diseases.^[^ ^7^, ^8^ ^]^ Accordingly, several experimental methods, such as T‐scan,^[^ ^9^ ^]^ TCR library screening,^[^ ^10^ ^]^ and tetramer‐associated TCR sequencing,^[^ ^11^ ^]^ have been developed to identify interactions between TCRs and epitopes. However, due to the inherent complexity of the TCR–epitope recognition mechanism, experimental detection and validation of these interactions are often time‐consuming, technically challenging, and costly, with a relatively low discovery rate.^[^ ^12^ ^]^ Therefore, the accurate and efficient recognition of TCR–epitope interactions remains a key computational challenge in modern immunology.

Numerous computational methods have been developed to predict TCR‐epitope binding.^[^ ^13^, ^14^ ^]^ These methods are primarily classified into two categories based on their prediction scope: epitope‐specific models and pan‐epitope models.^[^ ^15^ ^]^ Epitope‐specific models are designed to predict TCR‐epitope binding for a specific epitope and cannot generalize to novel epitopes, thus limiting their broader applicability.^[^ ^13^ ^]^ Examples of such models include TCRdist,^[^ ^16^ ^]^ TCRex,^[^ ^17^ ^]^ DeepTCR,^[^ ^18^ ^]^ TCRGP,^[^ ^19^ ^]^ and NetTCR‐2.^[^ ^20^ ^]^ In contrast, pan‐epitope models, which are not restricted to specific peptides, can predict TCR‐epitope binding for unseen epitopes by utilizing various deep learning techniques that offer greater generalization and flexibility. Notable examples include DLpTCR,^[^ ^21^ ^]^ an ensemble model that integrates sequence features from both TCRα and TCRβ chains; ImRex,^[^ ^22^ ^]^ which uses convolutional neural networks (CNNs) to capture physicochemical properties of TCR‐epitope interactions; and ERGO,^[^ ^23^ ^]^ which combines long short‐term memory (LSTM) networks with autoencoders to effectively model sequence dependencies and latent feature representations. Additionally, TEPCAM^[^ ^24^ ^]^ and ATM‐TCR^[^ ^25^ ^]^ incorporate attention mechanisms to improve prediction accuracy. Several methods, such as pMTNet,^[^ ^12^ ^]^ TEINet,^[^ ^26^ ^]^ and TITAN,^[^ ^27^ ^]^ employ pre‐trained models to enhance TCR‐epitope prediction performance. Furthermore, PanPep^[^ ^15^ ^]^ utilizes neural Turing machines (NTMs) to enhance model robustness, particularly in scenarios involving unseen epitopes.

Although recent models have achieved strong performance in specific settings, several key limitations hinder their broader application to TCR–epitope binding prediction. First, existing models often experience a significant performance decline when applied to unseen TCR or epitope sequences, limiting their utility in critical applications such as neoantigen discovery and immune repertoire profiling.^[^ ^28^ ^]^ Second, most models lack interpretability, offering limited insight into which residues in the TCR‐CDR3 and epitope drive binding specificity.^[^ ^12^ ^]^ Third, TCR–epitope binding is a distinct form of protein–protein interaction, where physicochemical properties such as charge, hydrophobicity, and flexibility critically shape structural and biochemical specificity.^[^ ^16^, ^29^ ^]^ Yet most existing models rely heavily on sequence‐level patterns,^[^ ^30^ ^]^ often oversimplifying or neglecting these determinants and failing to capture the multi‐scale, context‐dependent nature of TCR–epitope interactions. Together, these limitations underscore the urgent need for more generalizable and interpretable modeling frameworks that reflect the biochemical complexity of immune recognition.

To address these challenges, we propose DAISY, a biologically informed vision‐guided deep learning framework for robust and interpretable TCR–epitope binding prediction. DAISY introduces a multi‐level modeling paradigm grounded in immunological and biochemical principles. 1) At the feature representation level, DAISY innovatively integrates hierarchical physicochemical features by combining image‐like local interaction maps with global biochemical descriptors, thereby capturing both spatial specificity and global context essential for binding recognition. 2) At the architectural level, we introduce a biologically inspired, two‐step Condition‐Adaptive Fusion (CAF) module. The first step, inspired by TCR–CD28 co‐stimulation in immune activation,^[^ ^31^ ^]^ enables local and global features to jointly shape the fusion process via spatial–channel attention. The second step, motivated by cytokine‐guided differentiation,^[^ ^32^ ^]^ refines the fused representation under global guidance through condition‐aware optimization. 3) At the methodological level, DAISY unifies computer vision and immunological modeling through biologically inspired design, setting a methodological precedent and offering a scalable foundation for diverse immune modeling tasks.

We extensively evaluate DAISY across four representative recognition settings (Seen‐Pair, Unseen‐TCR, Unseen‐Epitope, and Unseen‐Pair), designed to mimic real‐world generalization scenarios. DAISY consistently outperforms state‐of‐the‐art models, achieving up to 11% improvement in the Area Under the Receiver Operating Characteristic Curve (ROC‐AUC) and 16% in the Area Under the Precision–Recall Curve (PR‐AUC) compared to the strongest baseline under the most challenging Unseen‐Pair setting. Additionally, DAISY uniquely supports end‐to‐end interpretability via Score‐CAM visualization^[^ ^33^ ^]^ of binding interfaces and is functionally validated through in silico alanine scanning,^[^ ^34^ ^]^ allowing biologically meaningful attribution at residue‐level resolution. Furthermore, we reveal the translational relevance of DAISY by bridging its computational predictions with key immunological and clinical outcomes: correlates with T‐cell clonal expansion, enables the identification of functional TCRs, and defines novel biomarkers that robustly forecast patient survival in immunotherapy, thereby highlighting its potential for personalized immunotherapy and vaccine design.

## 2 Results

### 2.1 DAISY Overview

**Figure 1:**
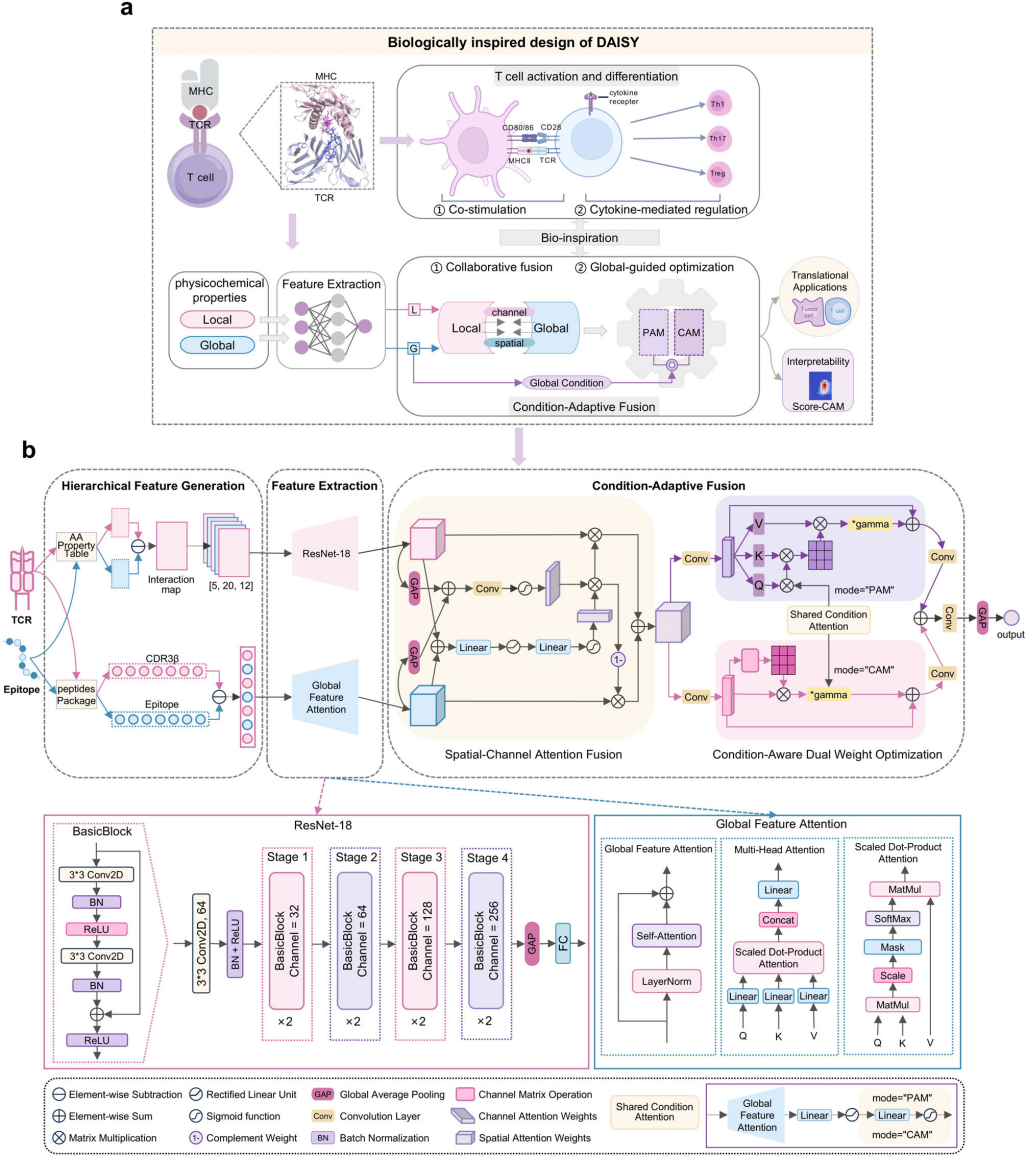
Overview of the DAISY framework. a) Biologically inspired design logic. DAISY's Condition‐Adaptive Fusion module is informed by T cell co‐stimulation and cytokine‐mediated regulation, implemented through a biologically inspired two‐step process. Step 1 performs collaborative fusion, where local and global features jointly guide spatial and channel attention. Step 2 applies global–guided optimization, where the fused representation is refined under global contextual guidance. DAISY also supports interpretability via Score‐CAM and demonstrates translational relevance in melanoma immunotherapy. b) Neural architecture of DAISY. The framework comprises four modules: 1) Hierarchical Feature Generation, which constructs local interaction maps and global difference vectors from TCR and epitope sequences; 2) Feature Extraction, which applies ResNet‐18 and Global Feature Attention to learn high‐level representations; 3) Condition‐Adaptive Fusion, which integrates local and global features through Spatial–Channel Attention Fusion and Condition‐Aware Dual Weight Optimization; and 4) Immunological Classification, which predicts the probability of TCR–epitope binding. Architectural details of ResNet‐18 and attention mechanisms are shown for clarity. Abbreviations: PAM, Position Attention Mechanism; CAM, Channel Attention Mechanism. Panels a created with BioRender.com.

DAISY is a biologically informed vision‐guided deep learning framework that accurately predicts TCR–epitope binding specificity across diverse generalization scenarios via Condition‐Adaptive Fusion of hierarchical physicochemical features. As shown in **Figure** **1**, the framework consists of four key modules:

1) Hierarchical Feature Generation Module: To capture both local and global determinants of TCR–epitope binding specificity, DAISY encodes each TCR–epitope pair through two complementary streams. Local interactions are modeled through interaction maps that encode pairwise physicochemical properties between residues, while global features are derived from whole‐sequence physicochemical properties.
2) Feature Extraction Module: Local interaction maps, treated as image‐like inputs, are processed through a ResNet‐based convolutional encoder^[^ ^35^ ^]^ to capture spatially structured binding patterns. In parallel, global physicochemical properties are processed by the Global Feature Attention module that dynamically reweights features based on biological relevance, enabling DAISY to prioritize informative biochemical signals.
3) Condition‐Adaptive Fusion Module (CAF): DAISY integrates local and global representations through a biologically inspired two‐stage process. In the first stage, inspired by the immunological co‐stimulation mechanism where TCR engagement and CD28 signaling jointly govern T cell activation,^[^ ^31^ ^]^ the Spatial‐Channel Attention Fusion submodule adaptively adjusts spatial and channel‐level weights to simulate biologically meaningful joint regulation of local and global features, thus preserving critical biological information while avoiding indiscriminate feature merging. In the second stage, inspired by cytokine‐mediated immune cell differentiation,^[^ ^32^ ^]^ the Condition‐Aware Dual Weight Optimization submodule further refines the fused representation by reweighting spatial and channel contributions based on global context, enabling the fused representations to be contextually optimized to capture biologically significant interactions.
4) Immunological Classification Module: The fused representation is fed into a classification module that outputs a continuous binding score between 0 and 1, reflecting the predicted likelihood of TCR–epitope binding.

Together, this unified design captures both spatially localized and globally contextualized patterns in TCR‐epitope interactions, enabling end‐to‐end interpretability and improving generalization beyond memorized sequence patterns across challenging scenarios.

### 2.2 DAISY Outperforms State‐of‐the‐Art Models Across Diverse Generalization Scenarios

To rigorously evaluate the predictive performance and generalizability of DAISY, we implemented a comprehensive validation framework that combined three types of fivefold cross‐validation (CV) on the training set (Tr‐TCR‐epitope) with evaluations on four carefully curated independent test sets. This dual strategy allowed assessment of both the model's robustness on familiar data and its generalization capacity under varying levels of novelty in the test scenarios. The following sections present a detailed comparison of model performance across both cross‐validation and independent testing settings.

The fivefold CV results demonstrated that DAISY consistently outperformed existing methods across all three validation scenarios (**Table** **1**). Specifically, in the regular CV setting, DAISY achieved a ROC‐AUC of 0.922 and a PR‐AUC of 0.936, significantly surpassing the top competitor, pMTNet,^[^ ^12^ ^]^ which obtained a ROC‐AUC of 0.857 and a PR‐AUC of 0.873. In the TCR‐grouped CV setting, which evaluates generalization to unseen TCR sequences, DAISY again achieved the highest ROC‐AUC of 0.915 and PR‐AUC of 0.934, substantially outperforming pretrained models such as TEINet,^[^ ^26^ ^]^ pMTNet,^[^ ^12^ ^]^ and TITAN‐PT.^[^ ^27^ ^]^ Under the more challenging epitope‐grouped CV setting, where models are evaluated on completely unseen epitopes, all baseline models experienced notable performance drops. Nevertheless, DAISY continued to maintain the best performance, with a ROC‐AUC of 0.800 and a PR‐AUC of 0.836.

**Table 1:** Performance comparison of DAISY with eight state‐of‐the‐art models under three cross‐validation settings. Each setting corresponds to a distinct generalization scenario: Seen‐Pair (regular fivefold CV), Unseen‐TCR (TCR‐grouped CV), and Unseen‐Epitope (epitope‐grouped CV). All results are reported as mean across five independent runs. The best results are highlighted in bold.

	Seen‐Pair		Unseen‐TCR		Unseen‐Epitope	
	ROC‐AUC	PR‐AUC	ROC‐AUC	PR‐AUC	ROC‐AUC	PR‐AUC
ERGO‐AE	0.822	0.506	0.824	0.510	0.640	0.319
ERGO‐LSTM	0.860	0.597	0.857	0.593	0.580	0.296
PISTE	0.744	0.797	0.768	0.825	0.760	0.793
TEIM	0.844	0.579	0.850	0.600	0.344	0.140
TEINet	0.721	0.743	0.707	0.737	0.517	0.571
TITAN‐PT	0.543	0.587	0.559	0.607	0.794	0.833
TITAN	0.547	0.582	0.551	0.590	0.602	0.763
pMTnet	0.857	0.873	0.845	0.866	0.784	0.812
DAISY	**0.922**	**0.936**	**0.915**	**0.934**	**0.800**	**0.836**

**Figure 2:**
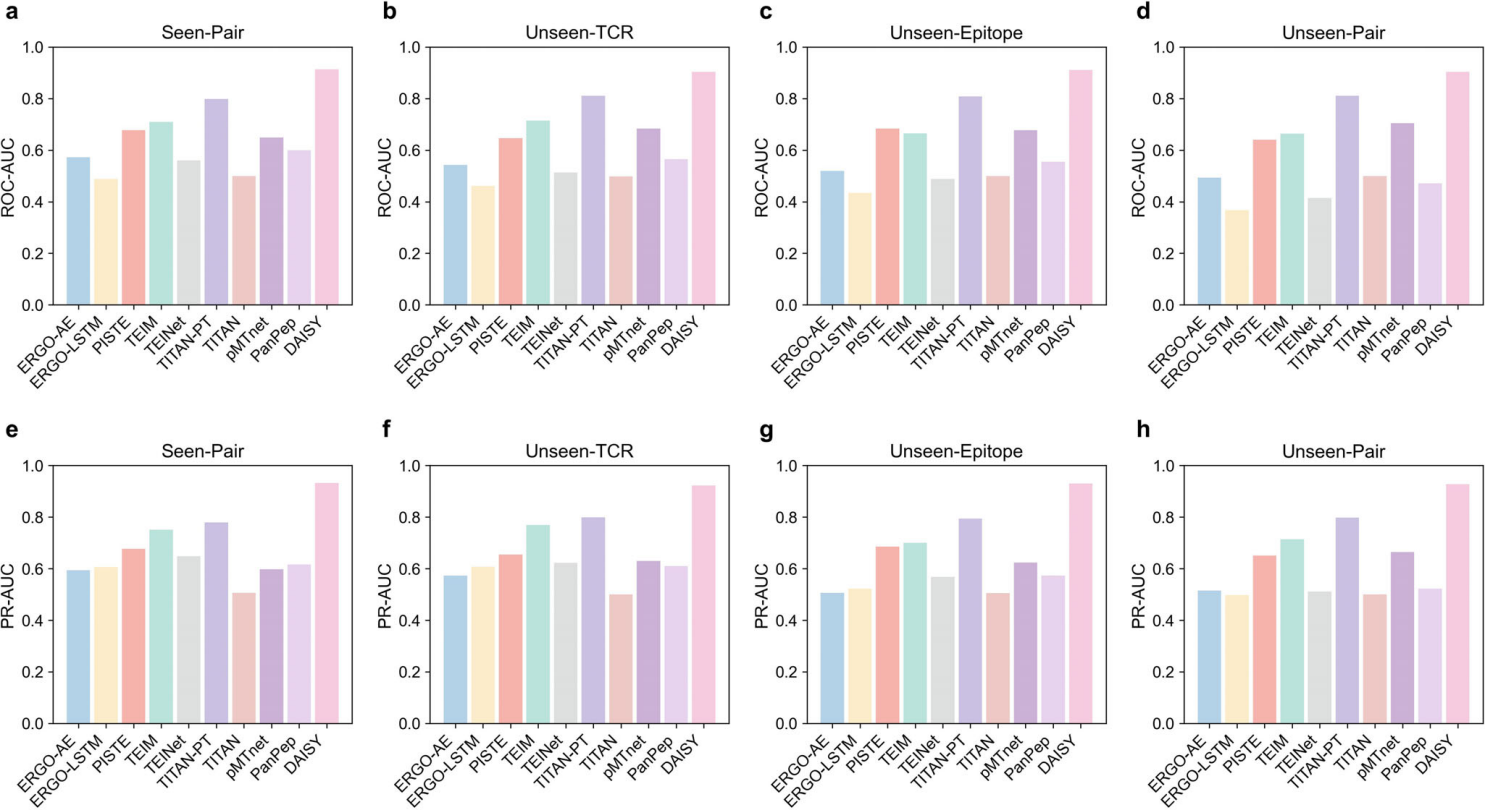
The performance comparison of DAISY and nine baseline models across four independent test sets. a, e) ROC‐AUC and PR‐AUC on the Seen‐Pair test set; b, f) ROC‐AUC and PR‐AUC on the Unseen‐TCR test set; c, g) ROC‐AUC and PR‐AUC on the Unseen‐Epitope test set; d, h) ROC‐AUC and PR‐AUC on the Unseen‐Pair test set. DAISY consistently outperforms all baselines across all evaluation scenarios, especially in settings involving completely novel TCRs or epitopes, demonstrating superior generalizability, and robustness.

To further assess generalization beyond the training domain, we evaluated all models on four independent datasets: Seen‐Pair, Unseen‐TCR, Unseen‐Epitope, and Unseen‐Pair. As shown in **Figure** **2**, DAISY consistently outperformed all models, demonstrating reliable generalization across all test sets. On the Seen‐Pair dataset, DAISY achieved a ROC‐AUC of 0.913 and a PR‐AUC of 0.933, while TITAN‐PT and PISTE^[^ ^36^ ^]^ followed with ROC‐AUCs of 0.798 and 0.678, and PR‐AUCs of 0.779 and 0.677, respectively, indicating weaker generalization. On the Unseen‐TCR dataset, all models except DAISY showed a slight performance drop compared to the Seen‐Pair dataset. Nevertheless, DAISY once again outperformed all competitors, including pretrained approaches (TEINet, TITAN‐PT), achieving the highest ROC‐AUC of 0.903 and PR‐AUC of 0.923. Similarly, the Unseen‐Epitope dataset led to a considerable decline in predictive performance for all baseline models. By contrast, DAISY continued to maintain the highest ROC‐AUC of 0.911 and PR‐AUC of 0.930, while the best competitor, TITAN‐PT, achieved a much lower ROC‐AUC of 0.808 and PR‐AUC of 0.794. PISTE and pMTNet were next in performance, with ROC‐AUCs of 0.684 and 0.677, and PR‐AUCs of 0.685 and 0.624, respectively. Moreover, the remaining models performed poorly, approaching random classification. On the most stringent Unseen‐Pair set, despite a slight decrease in ROC‐AUC and PR‐AUC from 0.911 to 0.903 and from 0.930 to 0.928, respectively, DAISY still demonstrated strong generalization to novel TCRs and epitopes. In comparison, TITAN‐PT ranked second with a ROC‐AUC of 0.811 and PR‐AUC of 0.798, while PISTE and TEIM performed significantly worse, further highlighting substantial performance gaps.

Overall, DAISY consistently demonstrated superior generalization ability across both cross‐validation and independent testing scenarios. In particular, it notably surpassed pretrained models such as TEINet, pMTNet, and TITAN‐PT, particularly in more challenging settings. This performance advantage was attributed to DAISY's capacity to effectively capture TCR–epitope binding patterns by integrating both local and global physicochemical properties, rather than relying on sequence memorization or similarity. As a result, DAISY can achieve robust generalization without the need for extensive pretraining data, underscoring the efficiency and efficacy of its design.

### 2.3 Module and Feature Attribution Analysis in DAISY

To evaluate the contribution of each individual component in DAISY, we conducted ablation studies under four distinct experimental settings. The evaluated model variants include the baseline ResNet‐18, versions with stepwise integration of the Global Feature Attention (GFA), Spatial‐Channel Attention Fusion (SCAF), Dual Attention Network (DANet), and Condition‐Aware Dual Weight Optimization (CADO), as well as the final fully integrated model DAISY. As shown in **Table** **2**, even the baseline ResNet‐18 achieved consistently strong performance across all evaluation metrics, with a ROC‐AUC of 0.866 and a PR‐AUC of 0.896, underscoring that local interaction features alone can form a solid foundation for TCR‐epitope binding prediction. Upon integrating the GFA module, performance notably improved, particularly in PR‐AUC (increasing to 0.913), highlighting the substantial contribution of global features in enhancing discriminative capability. Next, adding the SCAF module on top of the GFA‐enhanced model led to further improvements, demonstrating that spatial‐channel attention fusion effectively captures complementary local and global features to strengthen overall model performance. However, when the DANet module was directly integrated into the GFA‐enhanced model without incorporating the SCAF module, a performance decline was observed, with the ROC‐AUC dropping to 0.873. This suggests that the absence of SCAF may have led to misaligned attention interactions, where the uncoordinated integration of spatial and channel features introduced by DANet impaired the overall feature fusion process. In contrast, integrating the CADO module, which couples DANet with a Shared Condition Attention mechanism, into the ResNet‐GFA model led to a moderate recovery in performance, with ROC‐AUC and PR‐AUC rising to 0.887 and 0.914, respectively. This improvement highlights the crucial role of the Shared Condition Attention mechanism in aligning the spatial and channel attention pathways. By refining feature fusion, it also enhances the model's sensitivity to critical regions involved in TCR–epitope interactions. Interestingly, further integrating the SCAF module into the ResNet‐GFA‐DANet combination, forming the ResNet‐GFA‐SCAF‐DANet variant, resulted in a notable performance decline. This may be attributed to conflicting or redundant interactions introduced by multiple attention and fusion mechanisms operating in parallel without an explicit coordination strategy. Ultimately, DAISY, which synergistically integrated the ResNet backbone with GFA, SCAF, and the CADO module, achieved the best overall performance, demonstrating the robustness and effectiveness of the unified module integration. Moreover, evaluation under the Seen‐Pair, Unseen‐TCR, and Unseen‐Epitope settings (Tables S2–S4, Supporting Information) consistently demonstrates that DAISY outperformed all other variants, despite minor fluctuations in absolute performance scores. In conclusion, the ablation studies highlight the complementary contributions of each module and confirm the effectiveness of DAISY's design, which combines global and local features through condition‐aware integration.

**Table 2:** Performance comparison of DAISY and its ablated variants under the Unseen‐Pair setting, where the GFA, SCAF, DANet, and CADO modules are either removed or incrementally added. The best scores are highlighted in bold.

Model	ACC	ROC‐AUC	F1	PR‐AUC
ResNet‐18	0.809	0.866	0.803	0.896
ResNet‐GFA	0.826	0.887	0.818	0.913
ResNet‐GFA‐SCAF	0.820	0.904	0.818	0.931
ResNet‐GFA‐DANet	0.746	0.873	0.765	0.908
ResNet‐GFA‐CADO	0.804	0.887	0.802	0.914
ResNet‐GFA‐SCAF‐DANet	0.734	0.792	0.739	0.801
DAISY	**0.873**	**0.920**	**0.864**	**0.938**

**Figure 3:**
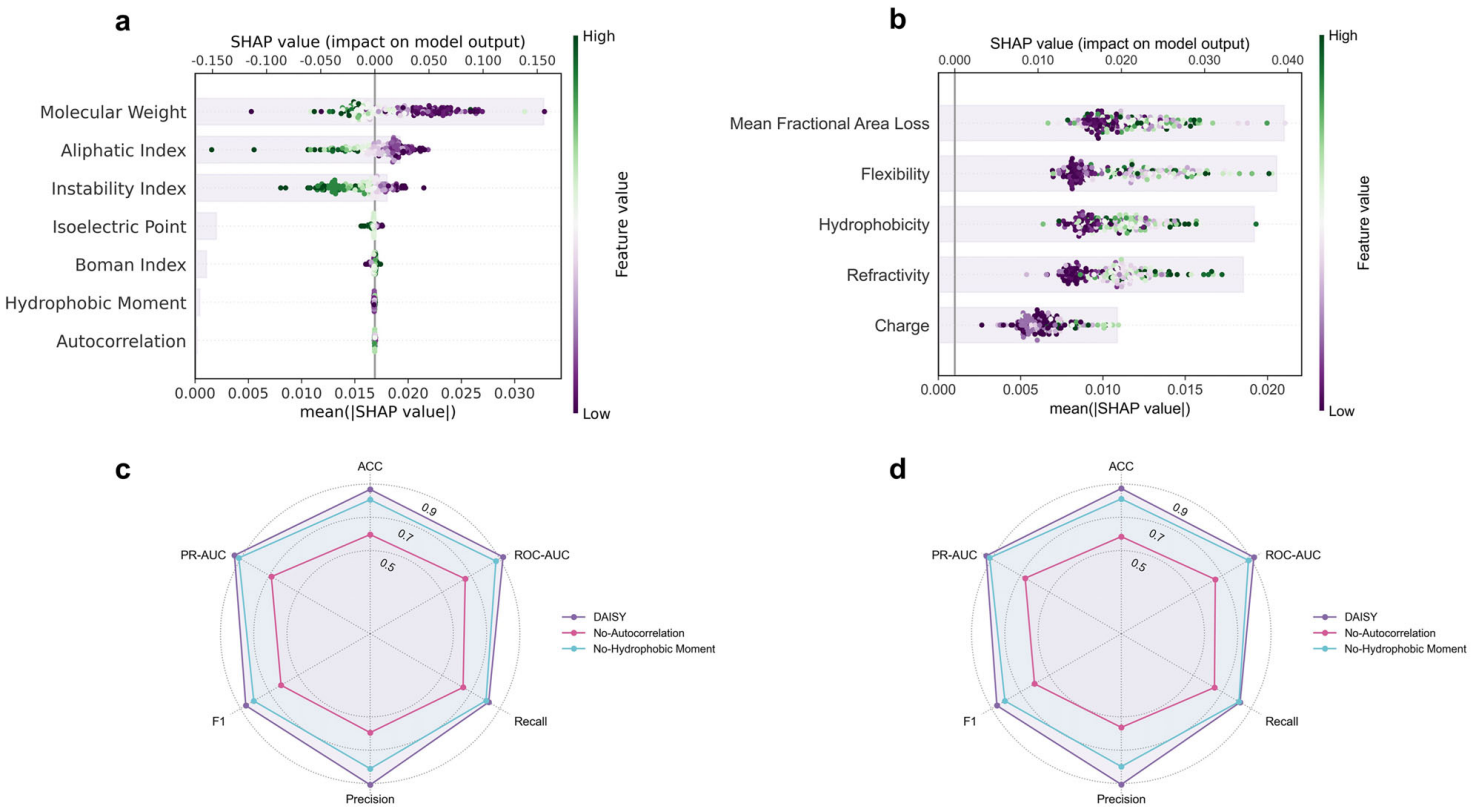
SHAP analysis and feature ablation results across various evaluation settings. a) SHAP summary plot of global physicochemical properties on positive TCR‐epitope pairs. b) SHAP summary plot of local physicochemical properties on positive TCR‐epitope pairs. Each dot represents the SHAP value of a single sample for the corresponding feature, with color indicating the feature value (from low to high, colored from purple to green). The accompanying bar chart displays the mean absolute SHAP value for each feature, reflecting its overall contribution to the model's prediction. c) Radar plot comparing the performance of DAISY with its ablated versions on the Unseen‐Epitope dataset. d) Radar plot comparing the performance of DAISY with its ablated versions on the Unseen‐Pair dataset. No‐Autocorrelation: model without autocorrelation; No‐Hydrophobic Moment: model without hydrophobic moment. Metrics include accuracy (ACC), ROC‐AUC, PR‐AUC, precision, recall, and F1‐score.

To comprehensively investigate the contribution of both global and local physicochemical properties to TCR‐epitope binding prediction, we employed SHAP (SHapley Additive exPlanations) for feature attribution analysis^[^ ^37^ ^]^ on both positive and negative samples. We first randomly selected 200 positive samples from a representative and well‐calibrated subset of all evaluation data. As illustrated in **Figure** **3**a, molecular weight, aliphatic index, and instability index displayed the highest mean SHAP values, indicating their dominant contribution to the model's prediction. In contrast, hydrophobic moment and autocorrelation consistently exhibited low SHAP values across most samples, suggesting a negligible influence on the model's decision‐making process. Local feature contributions are shown in Figure 3b, properties such as mean fractional area loss, flexibility, and hydrophobicity, which exhibited the highest mean SHAP values, played more prominent roles than charge and refractivity, highlighting the spatial specificity and biochemical compatibility essential for TCR‐epitope recognition. Moreover, the feature attribution patterns in negative samples exhibited partial overlap as well as divergence from those in positive samples, highlighting the model's adaptive reliance on context‐specific physicochemical properties (Figure S4, Supporting Information). In addition, given that the SHAP values of the global features hydrophobic moment and autocorrelation were close to zero in both positive and negative samples, we conducted feature ablation experiments across four independent test sets to rigorously evaluate whether these two global features were indeed redundant. For the Unseen‐Epitope and Unseen‐Pair sets (Figure 3c,d), removing autocorrelation resulted in a significant performance decline across all metrics, especially in ROC‐AUC and PR‐AUC. Similarly, the exclusion of hydrophobic moment led to a moderate yet consistent drop in performance. Interestingly, on the Seen‐Pair and Unseen‐TCR datasets, the ablation of hydrophobic moment did not impair performance and even yielded marginal improvements in certain metrics (Figure S5, Supporting Information). This unexpected outcome may be due to the lower task complexity of these settings, where reliance on dominant global features likely renders some complementary features redundant or even mildly interfering. Besides, from a biochemical perspective, the hydrophobic moment reflects the directional distribution of hydrophobic residues, particularly in helical or β‐structured segments. As an indicator of amphipathicity, it plays a key role at protein–protein interfaces such as TCR–epitope contacts, which may explain its selective importance under complex scenarios. In contrast, autocorrelation captures positional patterns of physicochemical properties, enabling recognition of structural motifs beyond the primary sequence. Its consistent performance impact underscores its essential role in modeling generalized interaction patterns. In summary, SHAP analysis, together with feature ablation, validated the design rationale of DAISY's global and local feature representations and demonstrated that low SHAP values do not inherently imply feature redundancy, particularly in diverse or challenging evaluation settings.

### 2.4 DAISY Identifies Key Binding Sites in TCR‐Epitope Interactions

**Figure 4:**
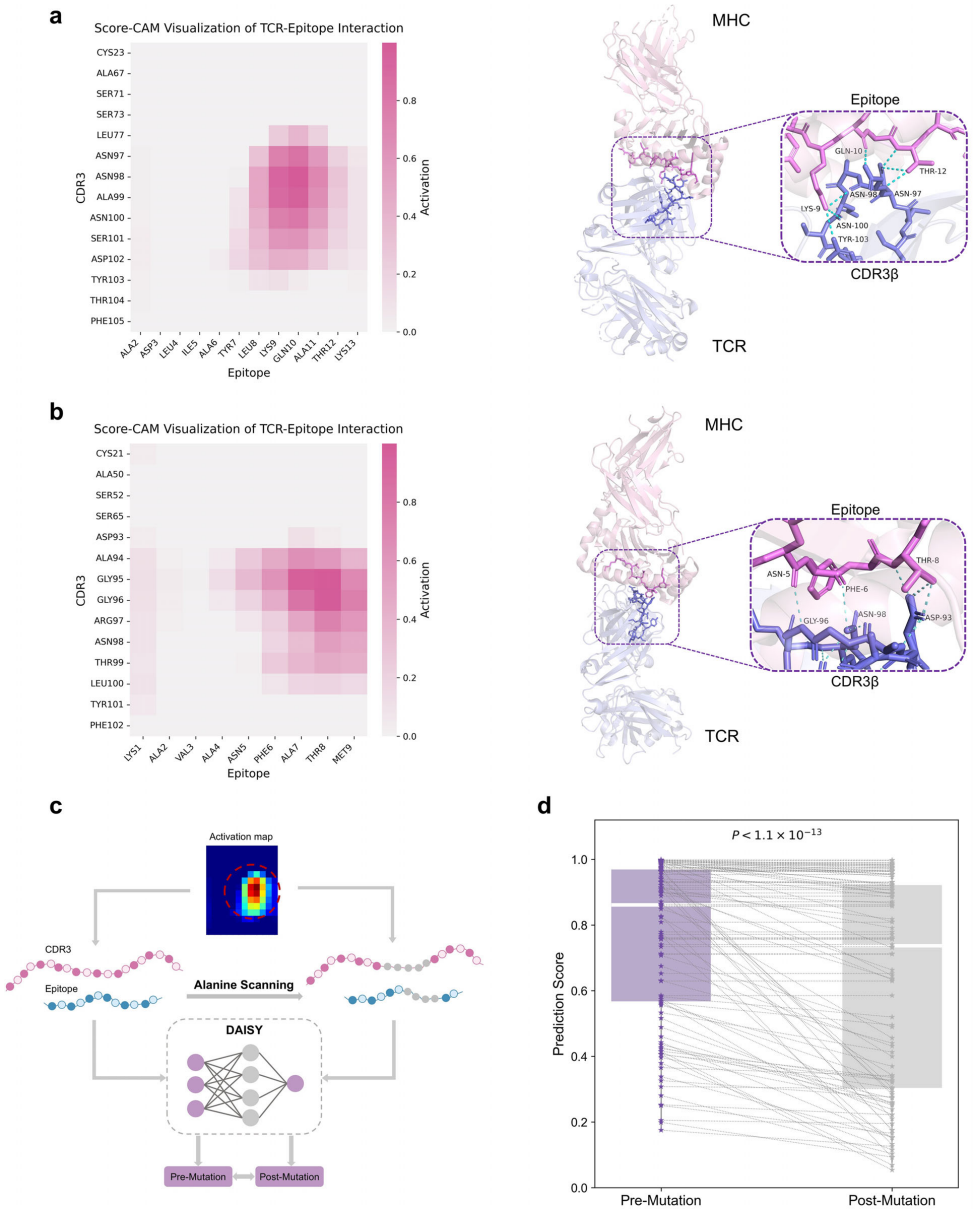
DAISY identifies structurally and functionally important sites in TCR–epitope interactions. a) Score‐CAM heatmap and PyMOL visualization of the TCR‐Epitope interaction (PDB ID: 3QIU). Left: Score‐CAM heatmap visualizing the interaction between the CDR3β and the epitope. Darker regions indicate higher activation values, corresponding to stronger model attention. Right: 3D structure of the TCR‐pMHC complex, highlighting key amino acid interactions between CDR3β (ASN‐97, ASN‐98, ASN‐100 and TYR‐103, colored in violet) and epitope (LYS‐9, GLN‐10, THR‐12, colored in slate). b) Score‐CAM heatmap and PyMOL visualization of the TCR‐Epitope interaction (PDB ID: 5M00). Left: Score‐CAM heatmap visualizing the interaction between the CDR3β and the epitope. Right: 3D structure of the TCR‐pMHC complex, highlighting key amino acid interactions between CDR3β (ASN‐5, ASN‐98, GLY‐96, colored in violet) and epitope (PHE‐6, THR‐8, and ASP‐93, colored in slate). c) Workflow of mutation analysis. Key residues identified from the activation map are subjected to alanine scanning within the CDR3 and epitope sequences. DAISY evaluates the interaction changes between pre‐ and post‐mutation sequences. d) Boxplot showing the average change in prediction scores before and after mutation across 112 TCR–pMHC complexes. A decrease in prediction scores was observed in 65.2% of the samples post‐mutation. The difference in prediction scores before and after mutation was statistically significant, as calculated by the Wilcoxon signed‐rank test (*P* < 1.1 × 10^−13^), indicating that mutations substantially affected DAISY's ability to identify key binding sites.

In addition to accurately predicting the likelihood of TCR‐epitope binding, DAISY can also identify key binding sites in TCR‐epitope interactions through Score‐CAM, an activation‐based visualization method that estimates the contribution of input regions to the prediction score without relying on gradients. By leveraging the image‐like properties of interaction maps, Score‐CAM^[^ ^33^ ^]^ can generate biologically meaningful heatmaps that highlight structurally important residues in TCR and epitope sequences, thereby further supporting model interpretability. Here, we collected 112 TCR–epitope pairs from STCRdab^[^ ^38^ ^]^ with three‐dimensional crystal structures available in the PDB database. Score‐CAM was then applied to the final convolutional outputs of DAISY to visualize the model's focus areas for these structures. In **Figure** **4**a,b, we show two TCR‐epitope crystal structures as examples by comparing the Score‐CAM heatmaps with the real binding structures visualized using PyMOL. In the first case (PDB ID: 3QIU), ASN‐97, ASN‐98, ASN‐100 and TYR‐103 in the CDR3β region of the TCR were found to interact with critical amino acids in the epitope presented by the MHC (LYS‐9, GLN‐10, THR‐12) through hydrogen bonds and polar interactions. Moreover, the aromatic ring of Tyr‐103 potentially contributes further stabilization via π‐π stacking interactions. As expected, the Score‐CAM mask generated by DAISY clearly highlighted the target regions, which align with the interaction residues identified in the PyMOL visualizations, despite the fact that DAISY did not utilize any structural data during training. In the second case (PDB ID: 5M00), key residues ASN‐5, ASN‐98, GLY‐96 in the CDR3β interacted with the epitope residues PHE‐6, THR‐8 and ASP‐93 through hydrogen bonds and other polar interactions. Similarly, these interacting residues were found to align with the activation regions in the Score‐CAM heatmap. The consistency between the Score‐CAM activation regions and the actual interaction sites demonstrated the effectiveness of Score‐CAM in evaluating DAISY's focus areas, confirming its utility for interpreting complex biological interactions.

To validate DAISY's sensitivity to binding sites, as well as further assess the versatility and robustness of Score‐CAM in capturing key binding sites, we performed in silico alanine scanning^[^ ^34^ ^]^ on the 112 TCR‐epitope pairs(Figure 4c). Alanine scanning is a site‐directed mutagenesis technique that systematically substitutes specific residues with alanine to evaluate their functional or binding contributions. For each sample, alanine substitutions were conducted at residues within the CDR3β and epitope regions that corresponded to Score‐CAM heatmap areas with activation values above a threshold. The results indicated that among 112 samples, 65.2% showed varying degrees of prediction score decline after mutation (Figure 4d). Furthermore, statistical analysis revealed a highly significant difference in prediction scores before and after mutation (*P* < 1.1 × 10^−13^), indicating that mutations significantly affected prediction scores in most samples, with the observed reductions strongly correlated with alterations at the CDR3–epitope binding interface. Some samples, particularly those with prediction scores close to 1, exhibited minimal changes after mutation, possibly due to the stability of their binding sites. Overall, these mutation experiments demonstrated that DAISY is sensitive to biologically meaningful structural changes and further validated its ability to identify critical TCR–epitope binding sites via Score‐CAM.

### 2.5 Applications of DAISY in Clinical Immunology

To demonstrate the potential clinical utility of DAISY, we applied it to several immunological applications: 1) DAISY qualitatively reveals antigen‐specific T cell clonal expansion, 2) DAISY enhances TCR sorting for personalized melanoma immunotherapy, and 3) DAISY improves prediction of clinical outcomes using functional biomarkers.

#### 2.5.1 DAISY Qualitatively Reveals Antigen‐Specific T Cell Clonal Expansion

Upon recognition of an antigen–HLA (pHLA) complex by the TCRs, T cells clonally expand to mount an effective immune response, with clonotypes of higher affinity for pHLA complexes being more prone to proliferate extensively.^[^ ^39^ ^]^ Here, we evaluated whether DAISY can qualitatively reflect this phenomenon by assessing the association between predicted binding scores and T cell clonal expansion.

**Figure 5:**
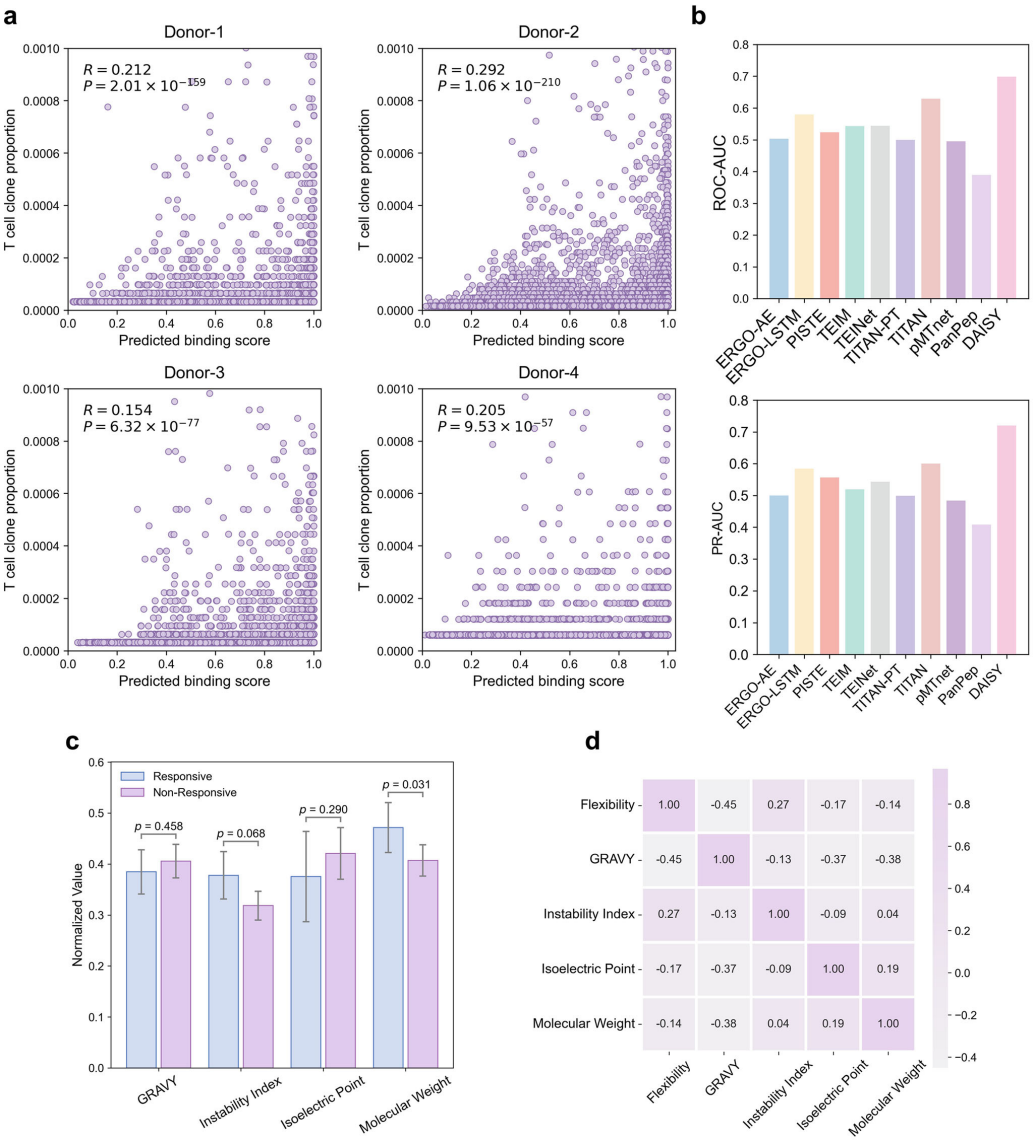
DAISY's predictions correlate with T‐cell clonal expansion and enhance functional TCR sorting. a) T cell clone proportion is positively correlated with predicted binding scores generated by DAISY in the 10x Genomics Chromium single‐cell immune profiling dataset. The Spearman correlation coefficient (*R*) and the corresponding two‐sided P values are indicated in each panel. b) Performance comparison of DAISY against state‐of‐the‐art models for immune‐responsive T‐cell sorting, evaluated by ROC‐AUC (top) and PR‐AUC (bottom) scores. c) Comparison of four physicochemical properties between responsive and non‐responsive T‐cell group. Values were min‐max normalized, and error bars represent 95% confidence intervals (bootstrap, 1000 iterations). The p‐value was calculated using the Mann–Whitney U test, with values shown above the bars. Note: Due to limited data availability, flexibility was excluded from significance testing but included in the correlation analysis. d) Heatmap of Pearson correlation coefficients between five physicochemical properties. Color intensity indicates the strength and direction of correlation. Most properties show weak correlations (|*r*| < 0.5), suggesting that the properties are relatively independent.

We used single‐cell TCR sequencing data from the 10x Genomics Chromium Single Cell Immune Profiling cohort, in which CD8^+^ T cells from four healthy donors were profiled against 44 pMHC complexes derived from cancer‐ and virus‐associated antigens. For each TCR clonotype, DAISY predicted the binding score to the 44 cognate pMHCs, and the highest score per clonotype was recorded. We then calculated the Spearman correlation coefficients between predicted binding scores and clonal fractions. As shown in **Figure** **5**a, we consistently observed a positive correlation between predicted binding scores and T cell clonal expansion across all donors. Clonotypes with higher predicted binding scores were more likely to be clonally expanded, consistent with biological expectations that TCRs with stronger binding affinities are more prone to clonal proliferation. These results indicate that DAISY reflects antigen‐specific clonal expansion of T cells and may serve as a qualitative indicator of clonal expansion.

#### 2.5.2 DAISY Enhances TCR Sorting for Personalized Melanoma Immunotherapy

Melanoma is a highly aggressive skin cancer characterized by a high mutational burden, making it an ideal candidate for adoptive cell transfer (ACT).^[^ ^40^, ^41^ ^]^ Recent clinical trials have highlighted the therapeutic promise of neoantigen‐specific T cell therapies, particularly for patients with metastatic melanoma who are refractory to immune checkpoint inhibitors (ICIs).^[^ ^42^, ^43^ ^]^ Nevertheless, improving the accuracy and efficiency of T cell selection remains a critical challenge for broad clinical translation. Here, we leveraged a benchmark dataset derived from the recent BNT221 trial,^[^ ^42^ ^]^ comprising experimentally validated TCR–neoantigen interactions specific to melanoma, spanning 34 distinct neoantigens. Negative samples were generated using reference TCR negative sampling, and all models were evaluated under the Unseen‐Epitope setting to simulate real‐world clinical scenarios. As shown in Figure 5b, DAISY outperformed all baseline methods, achieving a ROC‐AUC of 0.699 and a PR‐AUC of 0.720, both substantially higher than those of the best‐performing competitor, TITAN, which achieved a ROC‐AUC of 0.629 and a PR‐AUC of 0.600. These results indicate that DAISY is particularly effective at predicting immune‐responsive TCR‐neoantigen interactions, which is essential for improving T cell selection in personalized ACT therapies for melanoma.

Furthermore, we analyzed several physicochemical properties of neoantigens in immune‐responsive and non‐responsive TCR‐neoantigen interactions to assess their contribution to TCR sorting. A recent study investigated the VACCIMEL allogeneic melanoma vaccine,^[^ ^44^ ^]^ which induces T cell responses targeting neoantigens and tumor‐associated antigens in melanoma patients. In this study, we selected physicochemical properties relevant to our model based on data from four patients enrolled in the VACCIMEL melanoma vaccine trial. We then compared the physicochemical properties of immune‐responsive and non‐responsive neoantigens and assessed inter‐feature correlations. As shown in Figure 5c, molecular weight was the only feature that exhibited a statistically significant difference, with higher values observed in the immune‐responsive group (*p* = 0.031). By contrast, GRAVY, instability index, and isoelectric point did not show statistically significant differences (p‐values of 0.458, 0.290, and 0.068, respectively), indicating these features alone are insufficient to distinguish between immune response groups. In addition, correlation analysis (Figure 5d) revealed generally low inter‐feature correlations among these physicochemical properties, with most correlation coefficients below 0.4. Specifically, flexibility and GRAVY exhibited a moderate negative correlation (*r* = −0.45), while the correlations between other features remained weak, suggesting that these physicochemical properties are relatively independent and may exert complementary effects on immune responsiveness. Together, our results demonstrate the importance of complementary physicochemical features in modeling TCR–neoantigen interactions and further validate the rationale behind DAISY's multi‐feature design.

#### 2.5.3 DAISY Improves Prediction of Clinical Outcomes Using Functional Biomarkers

A comprehensive understanding of the interplay between neoantigens and the T‐cell repertoire is vital for assessing the efficacy of immune checkpoint blockade.^[^ ^45^ ^]^ While prior research has established Tumor Mutational Load (TML) and Neoantigen Load (NAL) as potential predictive biomarkers,^[^ ^46^, ^47^, ^48^, ^49^ ^]^ their utility is often limited as they merely quantify mutational burden without capturing its biological functionality. To overcome these limitations, we explored the predictive power of functional biomarkers that directly reflect neoantigen immunogenicity. We defined two such biomarkers: Immunogenic Neoantigen Load (INAL), an index adapted from previous work,^[^ ^36^ ^]^ representing the number of unique neoantigens recognized by high‐frequency T‐cell clonotypes, and Responsive T‐cell Clonotype Frequency (RTCF), defined as the cumulative frequency of all neoantigen‐reactive TCR clonotypes.

**Figure 6:**
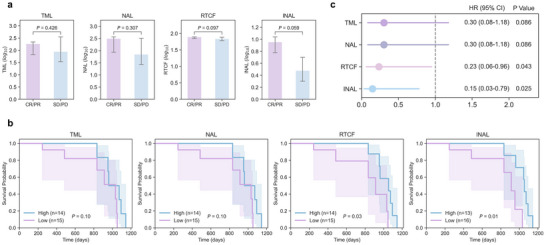
Functional neoantigen‐related biomarkers outperform conventional biomarkers in predicting clinical outcomes for advanced melanoma. a) Association of TML, NAL, RTCF, and INAL with clinical response. Biomarker levels are compared between patients who achieved a complete or partial response (CR/PR; *n* = 7) and those with stable or progressive disease (SD/PD; *n* = 21). Bars represent the median of log_10_‐transformed values, with error bars indicating the 90% confidence interval. P‐values were determined using a one‐sided Wilcoxon rank‐sum test. b) Kaplan‐Meier analysis of overall survival. Patients were stratified into High and Low groups based on the median value of each biomarker. The P‐value for the difference between curves was calculated using the log‐rank test. Shaded areas represent 95% confidence intervals. c) Univariate Cox regression analysis for OS. The forest plot displays the Hazard Ratios (HRs) for OS from a univariate Cox proportional hazards model, with each biomarker dichotomized as High versus Low relative to the median. Points indicate the HR, and horizontal lines represent the 95% confidence intervals (CI).

Here, we applied DAISY to a cohort of advanced melanoma patients treated with anti‐PD‐1 therapy to derive the functional indices INAL and RTCF, and compared the predictive power against the conventional biomarkers TML and NAL.^[^ ^50^ ^]^ We first investigated their association with short‐term clinical response. As presented in **Figure** **6**a, both functional biomarkers, INAL (*P* = 0.059) and RTCF (*P* = 0.097), showed a strong trend towards higher levels in the responder group (CR/PR; *n* = 7) compared to non‐responders (SD/PD; *n* = 21). In contrast, the conventional biomarkers TML and NA showed no such association. While the trend for functional biomarkers did not reach statistical significance, likely due to the limited sample size of the responder cohort, it pointed towards their superior relevance to therapeutic benefit. To assess their prognostic value for the more clinically meaningful endpoint of long‐term survival, we next performed Kaplan‐Meier analysis. This revealed that high levels of both functional biomarkers were significantly associated with improved overall survival (OS) (INAL, *P* = 0.01; RTCF, *P* = 0.03). Conversely, neither TML nor NAL was associated with OS, underscoring their limitations as prognostic tools in this cohort (Figure 6b). Univariate Cox analysis further quantified this survival advantage, confirming INAL (HR = 0.15) and RTCF (HR = 0.23) as significant protective factors (Figure 6c). Collectively, these results demonstrate the superior predictive value of DAISY‐derived functional biomarkers over conventional quantitative metrics. These two indices offer complementary insights: INAL quantifies the breadth of the dominant T‐cell response by counting unique immunogenic targets, whereas RTCF measures its total magnitude.

## 3 Discussion

DAISY presents a biologically informed vision‐guided framework for robust and interpretable TCR–epitope binding prediction. By unifying hierarchical physicochemical features via a biologically inspired, two‐step Condition‐Adaptive Fusion module, DAISY consistently outperforms state‐of‐the‐art models across four increasingly stringent generalization scenarios. In addition, DAISY provides deeper biological insights through two complementary avenues: 1) it uniquely supports end‐to‐end interpretability via Score‐CAM visualization of binding interfaces; and 2) significantly, it demonstrates profound translational relevance, as its predictions correlate with T‐cell clonal expansion, enable the identification of functional TCRs, and define novel biomarkers that robustly forecast patient survival in melanoma immunotherapy. More importantly, DAISY introduces a scalable modeling paradigm that bridges computer vision and immunoinformatics through biologically inspired modeling strategies, expanding the methodological repertoire for immune modeling and establishing a generalizable foundation for diverse applications in next‐generation immunoinformatics.

Although DAISY significantly outperformed existing models in this study, there are several areas for future improvement. Currently, DAISY focuses only on interactions involving the CDR3β chain of the TCR, primarily due to the limited availability of datasets containing both α‐ and β‐chain sequences. Similarly, the lack of comprehensive HLA typing data has restricted the integration of this crucial contextual information, although such data could provide valuable complementary insights. Furthermore, as more 3D crystal structure data become available, incorporating structural information is expected to enhance the precision of TCR–epitope binding predictions. Future work will focus on improving the model's biological relevance and predictive accuracy by integrating more comprehensive biological information, including dual‐chain TCR data, HLA information, and structural features, thereby broadening its translational potential in personalized immunotherapy.

## 4 Experimental Section

### Design of DAISY

DAISY was proposed, a biologically informed vision‐guided deep learning framework tailored to predict TCR–epitope interactions across four distinct settings: Seen‐Pair, Unseen‐TCR, Unseen‐Epitope, and Unseen‐Pair. The overall framework consisted of four key modules: the Hierarchical Feature Generation Module, Feature Extraction Module, Condition‐Adaptive Fusion Module (CAF), and Immunological Classification Module. This systematic architecture significantly enhanced the accuracy and reliability of TCR–epitope interaction predictions, making it a powerful tool for binding prediction.

The DAISY framework is shown in Figure 1, and the following sections provide a detailed description of each module, including its components, functionality, and contribution to the overall architecture.

### Design of DAISY‐Hierarchical Feature Generation Module

The Hierarchical Feature Generation Module was designed to generate both local and global features by calculating the differences in physicochemical properties between the input TCR‐CDR3 and epitope sequences. This module captured the diversity and complexity of TCR‐epitope interactions, which vary across different epitopes and TCR sequences, and comprises two main components: Local Interaction Feature Generation and Global Feature Calculation.

1) Local Interaction Feature Generation

To capture the fine‐grained residue‐level interaction patterns between CDR3s and epitopes, local interaction maps were construct based on five selected physicochemical properties of amino acids: hydrophobicity, refractivity, charge, flexibility, and mean fractional area loss. Specifically, each amino acid was represented by a 5‐D physicochemical property vector, forming a feature matrix as follows:

(1) $$A=\left\{A_{1},A_{2},\text{…},A_{n}\right\},\;A_{i}\in R^{5}$$

where *n* denotes the sequence length, zero‐padded to 20 for CDR3 and 12 for epitope. The interaction tensor $I_{f}\in R^{5\times 20\times 12}$ was then calculated as the absolute pairwise differences between the physicochemical property vectors of the CDR3 and epitope:

(2) $$I_{f}\left[i,j\right]=\left|A_{\mathrm{CDR}3}\left[i\right]-A_{\mathrm{Peptide}}\left[j\right]\right|,\;i\in \left\{0,1,\text{…},19\right\},\;j\in \left\{0,1,\text{…},11\right\}$$

The interaction tensor **I**_f_, in which each channel corresponds to one physicochemical property, was subsequently used as input to the Local Feature Extraction Module.

2) Global Feature Calculation

In addition to the local interaction information, the global physicochemical properties of CDR3 and epitope sequences capture sequence‐wide features that play a crucial role in binding stability and affinity, thus providing essential context for modeling the overall interaction. Specifically, for each CDR3 and epitope sequence, seven global physicochemical properties were calculated: isoelectric point (pI), molecular weight, instability index, aliphatic index, Boman index, hydrophobic moment, and autocorrelation. Let $G_{\mathrm{TCR}}=\left[g_{1},g_{2},\text{…},g_{7}\right]$ and $G_{\mathrm{Peptide}}=\left[g_{1}^{\prime },g_{2}^{\prime },\text{…},g_{7}^{\prime }\right]$ represent the global feature vectors for the CDR3 and epitope, respectively. The difference vector between these global features is computed as follows:

(3) $$G_{\mathrm{Diff}}=G_{\mathrm{TCR}}-G_{\mathrm{Peptide}}$$

The global difference vector, $G_{\mathrm{Diff}}\in R^{1\times 7}$, quantified the dissimilarities between the global physicochemical properties of the CDR3 and epitope sequences and serves as a key input to the Feature Extraction module.

### Design of DAISY‐Feature Extraction Module

The Feature Extraction Module was responsible for extracting both local and global features from the CDR3‐epitope interaction maps and global difference vectors, respectively, to capture complex interaction patterns and to enhance feature representations. This module consists of two main components: Local Feature Extraction and Global Feature Enhancement.

1) Local Feature Extraction

To capture more complex interaction patterns, a deep residual network (ResNet) was utilized to extract high‐level local features from the interaction maps.^[^ ^35^ ^]^ The ResNet architecture was composed of multiple residual blocks (BasicBlock), which effectively mitigates the vanishing gradient problem, thereby ensuring stable training and efficient feature extraction. In this study, the network consisted of four residual blocks, with an initial convolution kernel size of 3 × 3. The local feature extraction process is then performed using ResNet as follows:

(4) $$F_{L}=\mathrm{ResNet}\left(I_{f}\right)$$

here, $F_{L}\in R^{\mathrm{batchsize}\times 256\times H\times W}$ represents the output of the local feature extraction module, where 256 is the number of channels, and H and W correspond to the height and width of the spatial dimensions of the feature map.

2) Global Feature Enhancement

To enhance the representation of global features and facilitate their integration with local features, a Global Feature Attention (GFA) mechanism was emploted to enable the model to focus on the most relevant features for accurate TCR‐epitope binding prediction. First, the global feature vectors we normalized using Layer Normalization to ensure consistent value distribution:

(5) $$G_{\mathrm{Norm}}=\mathrm{LayerNorm}\left(G_{\mathrm{Diff}}\right)$$

where $G_{\mathrm{Norm}}\in R^{1\times 7}$ retains the same shape as $G_{\mathrm{Diff}}$.Then, a self‐attention mechanism^[^ ^51^ ^]^ was applied to compute an attention‐refined feature vector, $O_{A}$. This vector was then added to $G_{\mathrm{Norm}}$ through a residual connection to obtain the final global feature representation as follows:

(6) $$F_{G}=O_{A}+G_{\mathrm{Norm}}$$

This residual operation preserved the normalized global context while integrating the refined attention information. The resulting global feature representation, $F_{G}$, was subsequently processed to align with the spatial dimensions of the local features. Let **F**_G_ denote the aligned global feature tensor, which was then used for integration into the CAF module.

### Design of DAISY‐Condition‐Adaptive Fusion Module (CAF)

To overcome the limitations of traditional feature concatenation, a novel two‐stage fusion framework was proposed: Condition‐Adaptive Fusion Module. In the first stage, Spatial‐Channel Attention Fusion dynamically integrates local, and global features. In the second stage, Condition‐Aware Dual Weight Optimization performs joint spatial and channel attention refinement on the fused representation, guided by global context.

1) Spatial‐Channel Attention Fusion

In this module, both global and local feature representations collaboratively guide the fusion process. The global feature vector provided contextual guidance that, together with local features, dynamically modulated the attention weights for both channel and spatial fusion. This joint attention mechanism enabled the model to adaptively control the contribution of each feature source based on their contextual relevance.

Spatial Attention Calculation: To capture spatial dependencies across feature maps, a 7 × 7 convolution was applied to the sum of local and global features. A Sigmoid activation was then used to compute the spatial attention weights:

(7) $$W_{\mathrm{spatial}}=\sigma \left({\mathrm{Conv}}_{7\times 7}\left(F_{L}+F_{G}\right)\right)$$

where σ denotes the Sigmoid function, ensuring the attention weights fall within the range [0, 1].

Channel Attention Calculation: To capture the global importance of each feature channel, Global Average Pooling (GAP) was first applied to both the local and the global features. These pooled features were summed and passed through a fully connected layer, followed by a Sigmoid activation function to compute the channel attention weights:

(8) $$W_{\mathrm{channel}}=\sigma \left(\mathrm{FC}\left(\mathrm{GAP}\left(F_{L}\right)+\mathrm{GAP}\left(F_{G}\right)\right)\right)$$

Adaptive Fusion: The final fused feature map was obtained by combining local and global features using the computed spatial and channel attention weights. Specifically, the attention‐modulated local and global features were then combined via element‐wise weighted summation as follows:

(9) $$\begin{array}{cc} W_{\mathrm{fused}} & =W_{\mathrm{spatial}}⊙W_{\mathrm{channel}} \end{array}$$

(10) $$\begin{array}{cc} F_{\mathrm{fused}} & =W_{\mathrm{fused}}⊙F_{L}+\left(1-W_{\mathrm{fused}}\right)⊙F_{G} \end{array}$$

where ⊙ denotes element‐wise multiplication, and **1** is a tensor of ones broadcast to match the feature map dimensions. The fused feature map, $F_{\mathrm{fused}}\in R^{\mathrm{batchsize}\times 256\times H\times W}$, is then passed to the next module for further attention‐based optimization.

2) Condition‐Aware Dual Weight Optimization

To further enhance the fusion process, this module refines the fused feature map by generating condition‐aware attention weights derived from the enhanced global feature representation. By guiding the Position Attention Mechanism (PAM) and Channel Attention Mechanism (CAM),^[^ ^52^ ^]^ these condition weights optimize the fusion process along both spatial and channel dimensions, thereby improving the model's adaptive sensitivity to critical regions in TCR–epitope interactions.

Shared Condition Attention: Leveraging the enhanced global feature representation $F_{G}$, the PAM and CAM condition weights were computed through a shared network pipeline followed by separate branches for PAM and CAM as follows:

(11) $$\begin{array}{cc} W_{\mathrm{PAM}} & =\sigma \left(f_{\mathrm{PAM}}\left(f_{S}\left(F_{G}\right)\right)\right) \end{array}$$

(12) $$\begin{array}{cc} W_{\mathrm{CAM}} & =\sigma \left(f_{\mathrm{CAM}}\left(f_{S}\left(F_{G}\right)\right)\right) \end{array}$$

here, *f*_S_ is a shared two‐layer MLP with ReLU activations that process the $F_{G}$; *f*_CAM_ and *f*_PAM_ are the respective networks responsible for computing the condition weights for channel and spatial attention.

PAM Optimization: The PAM module enhanced spatial feature representation by modulating the fused feature map **F**_fused_, using $W_{\mathrm{PAM}}$. Specifically, the **F**_fused_ was first transformed via $W_{\mathrm{PAM}}$ to generate the query representation, while the key K and value V were obtained by applying separate 1×1 convolutions to the **F**_fused_. These components were then passed into the PAM attention mechanism to compute the spatially‐aware output:

(13) $$O_{\mathrm{PAM}}=\mathrm{PAM}\left(F_{\mathrm{fused}}⊙W_{\mathrm{PAM}},\;K,\;V\right)$$

CAM Optimization: Similarly, the CAM module refined channel‐wise representations by applying $W_{\mathrm{CAM}}$ to the **F**_fused_. Specifically, the **F**_fused_ was first transformed via $W_{\mathrm{CAM}}$ to generate the query representation, while the key K and value V are obtained by reshaping the input. The channel‐aware output is computed as follows:

(14) $$O_{\mathrm{CAM}}=\mathrm{CAM}\left(F_{\mathrm{fused}}⊙W_{\mathrm{CAM}},\;K,\;V\right)$$

Dual‐Attention Fusion: Finally, the outputs of both the PAM and CAM modules were aggregated to form the final feature representation:

(15) $$F_{\mathrm{final}}=O_{\mathrm{PAM}}+O_{\mathrm{CAM}}$$

This fusion combined spatial and channel‐wise attention refinements, enabling adaptive focus on key regions for more accurate prediction.

### Design of DAISY‐Immunological Classification Module

Based on the fused and optimized features, this module performed the final classification step to determine whether the TCR‐epitope interaction was binding or non‐binding. The final feature map **F**_final_ was first transformed into a compact vector, $v_{\mathrm{final}}$, via GAP:

(16) $$v_{\mathrm{final}}=\mathrm{GAP}\left(F_{\mathrm{final}}\right)$$

The resulting vector was then passed through a fully connected (FC) layer, followed by a Softmax activation to obtain the final classification output:

(17) $$O_{\mathrm{cls}}=\mathrm{Softmax}\left(\mathrm{FC}\left(v_{\mathrm{final}}\right)\right)$$

here, the Softmax function converts the output logits into a probability distribution over the two classes, with $O_{\mathrm{cls}}$ indicating the predicted likelihood of TCR‐epitope binding.

### Data Curation

To train the model, experimentally verified TCR–epitope pairs we collected as positive samples from four publicly available datasets: IEDB,^[^ ^53^ ^]^ PIRD,^[^ ^54^ ^]^ McPAS‐TCR,^[^ ^55^ ^]^ and VDJdb.^[^ ^56^ ^]^ For each database, only zHomo sapiens‐specific records, epitopes presented by human MHC I molecules and TCRs featuring only the CDR3β chain were retained, as these were critical for TCR–epitope binding specificity. The curated training set (termed Tr‐TCR‐epitope) comprises 70,469 positive TCR–epitope pairs, including 64,166 unique CDR3β sequences and 127 unique epitopes.

To rigorously evaluate model generalizability, four independent test sets were constructed based on the pMTnet test set, the PanPep test set, and an additional subset held out from the training data. To prevent data leakage and ensure unbiased evaluation, all exact TCR–epitope pairs present in the training dataset were strictly excluded. The construction strategy was designed to cover a spectrum of generalization scenarios, ranging from partially seen TCRs or epitopes to cases where both the TCR and epitope were entirely novel. The details are as follows:

Seen‐Pair: Serves as the base test set, evaluating generalization to previously observed TCRs or epitopes while ensuring that no exact TCR–epitope pairs overlap with the training data. This set contained 792 positive samples, including 370 unique epitopes and 447 unique TCRs.

Unseen‐TCR: Derived from Seen‐Pair by removing any TCRs overlapping with the training data. It contained 658 positive samples with 296 unique epitopes and 415 unique TCRs, thereby testing the model's ability to recognize novel TCRs.

Unseen‐Epitope: Derived from Seen‐Pair by excluding all epitopes observed in the training data. It comprised 580 positive samples, covering 341 unique epitopes and 256 unique TCRs, thus evaluating generalization to novel epitopes.

Unseen‐Pair: Derived from Seen‐Pair by simultaneously removing all TCRs and epitopes that overlapped with the training data. This dataset contains 449 positive samples with 267 unique epitopes and 226 unique TCRs, representing the strictest test case where both the TCRs and epitopes were entirely novel.

To generate negative samples, reference TCR negative sampling was adopted. From 60 million CDR3β sequences obtained from the peripheral blood of 587 healthy individuals without known antigen exposure, 10,000 sequences (termed small‐healthy‐TCR) were randomly selected.^[^ ^57^, ^58^ ^]^ Each negative pair was created by mismatching a positive epitope with a randomly selected CDR3 from the reference set. All training and test datasets were balanced with a 1:1 ratio of positive and negative samples.

### Performance Evaluation and Benchmarking

Baseline models: To assess the generalizability of DAISY, nine supervised models were selected as baselines. ERGO‐LSTM and ERGO‐AE were two variants of ERGO,^[^ ^23^ ^]^ based on LSTM and Autoencoder, respectively. For TITAN, both its scratch‐trained and pretrained version (TITAN‐PT) were evaluated.^[^ ^27^ ^]^ TEINet was tested using its pretrained model.^[^ ^26^ ^]^ Since this study only utilized TCR and epitope data, PISTE and pMTNet^[^ ^12^ ^]^ were evaluated using its 2nd‐order version.^[^ ^36^ ^]^ Additionally, for TEIM, the ‘seq inference’ model was employed.^[^ ^59^ ^]^ PanPep^[^ ^15^ ^]^ was evaluated in its few‐shot and zero‐shot configurations. All baseline models were retrained and evaluated using the same datasets as DAISY, following their respective default configurations.

Benchmark testing: To comprehensively evaluate model performance, three distinct fivefold cross‐validation (CV) strategies were applied to the training dataset, followed by evaluation on four independent test sets. The three fivefold CV strategies were designed to systematically evaluate different aspects of generalization: 1) a regular fivefold CV, where epitopes or TCRs could overlap between folds, but exact

TCR–epitope pairs were disjoint, evaluating generalization to new pairings; 2) an epitope‐grouped fivefold CV, in which epitopes were held out across folds, testing generalization to entirely novel epitopes; 3) a TCR‐grouped fivefold CV, in which no TCRs were shared across folds, measuring generalization to unseen TCRs. To ensure fair comparisons between models, all cross‐validation experiments were conducted using the same random seed. Subsequently, the model achieving the highest ROC‐AUC (Area Under the Receiver Operating Characteristic Curve) in the regular fivefold CV was selected for final evaluation on the four independent test sets.

Performance evaluation metrics: The performance was evaluated using ROC‐AUC (Area Under the Receiver Operating Characteristic Curve) and PR‐AUC (Area Under the Precision‐Recall Curve), which assessed the overall effectiveness of the binary classifier. ROC‐AUC, which plotted the TPR (true positive rate) against the FPR (false positive rate) at various decision thresholds, assesses the model's ability to distinguish between positive and negative samples. PR‐AUC, which plotted precision against recall, evaluated the trade‐off between these metrics and was particularly important for imbalanced datasets.

### Model Training and Optimization

To train DAISY, the AdamW optimizer was used with an initial learning rate of 0.005, a batch size of 256, and a maximum of 100 epochs. The model was trained to minimize the cross‐entropy loss function:^[^ ^60^ ^]^

(18) $$L=-\frac{1}{N}\sum_{i=1}^{N}\left[y_{i}\log\left(p_{i}\right)+\left(1-y_{i}\right)\log\left(1-p_{i}\right)\right]$$

where *p*_*i*_ is the predicted probability of class 1 for the *i*‐th sample, and *y*_*i*_ ∈ {0, 1} is the corresponding ground‐truth label.

To prevent overfitting, a learning rate decay strategy was applied with the ReduceLROnPlateau scheduler, which reduced the learning rate by a factor of 0.3 if the validation loss did not improve for two consecutive epochs. Early stopping was also implemented, halting training after six consecutive epochs without improvement.

For hyperparameter tuning, the Tr‐TCR‐epitope dataset was split into 90% for training and 10% for validation. The best hyperparameters were selected based on the highest ROC‐AUC and PR‐AUC scores on the validation set.

### Data Curation for TCR Sorting and Neoantigen Feature Analysis in Melanoma Immunotherapy

Benchmark Dataset from the BNT221 Trial: To evaluate DAISY in clinically relevant settings, a benchmark dataset derived from the BNT221 clinical trial in metastatic melanoma was utilized.^[^ ^42^ ^]^ This dataset reflects real‐world immunotherapy scenarios in which patient‐specific neoantigens were unseen during training, making evaluation under the Unseen‐Epitope setting representative of realistic therapeutic conditions. The dataset comprised experimentally validated TCR‐neoantigen interactions involving a total of 34 identified neoantigens. Negative samples were generated through reference TCR‐negative sampling to simulate non‐reactive interactions. DAISY against several existing methods were benchmarked, including ERGO‐AE, ERGO‐LSTM, PISTE, TEIM, TEINet, and TITAN. DAISY was applied in a zero‐shot manner, without any fine‐tuning on melanoma‐related data prior to evaluation.

The VACCIMEL Trial Dataset: To investigate the physicochemical properties of neoantigens relevant to immune responsiveness, data was collected from the VACCIMEL melanoma vaccine trial,^[^ ^44^ ^]^ which evaluated T‐cell responses to both neoantigens and tumor‐associated antigens (TAAs) in melanoma patients. Specifically, data from four patients enrolled in the trial were analyzed to quantify key physicochemical features of the identified neoantigens, including molecular weight, GRAVY (grand average of hydropathy), instability index, isoelectric point, and flexibility. The dataset was pre‐processed to normalize all features and ensure comparability for predictive modeling.

### Statistical Analysis

All statistical analyses were performed in Python (v3.9) with the SciPy (v1.7.3) and lifelines (v0.27.0) packages. The specific statistical tests used for each analysis, including details on group comparisons, correlations, and survival modeling, were described in the corresponding figure legends. Sample sizes (n) were also provided in the figure legends. Across all analyses, a *P* < 0.05 was considered statistically significant.

## Conflict of Interest

The authors declare no conflict of interest.

## Author Contributions

Y.Y. conceived and designed the study, developed the algorithm, performed the experiments, analyzed the data, and drafted the manuscript. J.C., Y.Z. and Y.C. revised the manuscript. Y.F. and C.Z. contributed to methodology design and validation. J.L. and Y.L. contributed to data curation and software implementation. Z.F. performed biological correlation analyses and assisted with data interpretation. D.W. supervised the study and provided overall guidance. All authors reviewed and approved the final version of the manuscript.

## Supporting information

Supporting Information

## Data Availability

The full datasets and source code for DAISY are publicly at https://github.com/yy‐peri/DAISY.
