# Supplementary material for: A Biologically Informed Vision‐Guided Framework for Interpretable T Cell Receptor–Epitope Binding Prediction
Source: Adv Sci (Weinh). 2025 Nov 7;13(4):e12544. doi: 10.1002/advs.202512544 (PMC12822471; doi:10.1002/advs.202512544)
Supplement: Supplementary file 1 — Supporting Information [file ADVS-13-e12544-s001.pdf]

A Biologically Informed Vision-Guided Framework for Interpretable T Cell Receptor–Epitope Binding Prediction

*Yajing Yuan, Junwei Chen, Yufang Zhang, Yitian Fang, Zhongcheng Fang, Yanyi Chu, Jiayi Li, Chen Zhang, Yuzhe Li, Dongqing Wei**

Supplementary Notes

1. Limitations of traditional sequence-based methods

Traditional approaches to TCR–epitope interaction prediction primarily rely on sequence-based modeling[1], using raw amino acid sequences as input to deep learning frameworks. While effective to some extent, these methods often treat residues as discrete symbols, neglecting the physicochemical and structural properties that underpin molecular recognition. As a result, key factors such as charge distribution[2], hydrophobicity, conformational flexibility, and thermodynamic stability are overlooked. Moreover, these models struggle to differentiate between residues that are similar in physicochemical nature[3] but differ in context or position, limiting their generalization to unseen TCRs or epitopes. To overcome these limitations, we incorporate a range of local and global physicochemical properties, allowing the model to learn from both residue-level interactions and sequence-level biochemical properties essential for immune specificity.

2. Biological rationale of DAISY's condition-adaptive feature fusion strategy

DAISY integrates local and global biochemical representations in a biologically meaningful manner, drawing parallels to well-established T cell activation mechanisms.

Stage 1: Co-regulated Fusion (Inspired by TCR-CD28 Co-stimulation): T cells require dual signals for full activation: TCR engagement (antigen-specific) and CD28 co-stimulation (contextual)[4]. Analogously, we designed a module where local interaction maps and global descriptors co-regulate feature integration via Spatial-Channel Attention. This ensures meaningful interaction between molecular specificity

and contextual background.

Stage 2: Context-Driven Refinement (Inspired by Cytokine Modulation): Once activated, T cells are further shaped by cytokines [5](e.g., IL-2, IL-12), which tailor their functional fate based on immune context. In our framework, this is mirrored by a Condition-Aware Dual Weight Optimization module that dynamically refines fused features under the guidance of global descriptors, analogous to cytokine-tuned cell differentiation.

Note: These biological processes serve as conceptual anchors, not literal computational modeling.

3. DAISY Architecture Summary

The TCR-epitope interaction is a distinct type of protein-protein interaction (PPI), in which molecular physicochemical properties such as charge, hydrophobicity, and flexibility play a critical role in determining binding specificity. Unlike existing sequence-based methods, which often overlook the significance of these molecular properties, DAISY innovatively integrates both global and local physicochemical properties of TCR and epitope, generating interaction maps and global feature vectors. Notably, this unique form of feature generation guides the selection of the feature extraction module. Specifically, local interaction maps can be represented as image-like data, making them suitable for processing by ResNet[6], a deep convolutional neural network widely used in computer vision due to its strong ability to capture intricate spatial features. For global feature extraction, we incorporate the self-attention mechanism, a technique that has achieved notable success in natural language processing[7] (NLP). This mechanism dynamically assigns varying weights to global features, enabling the model to identify key biological signals within the global feature vector. To effectively integrate the extracted features, we develop the novel Condition-Adaptive Fusion Module (CAF), which enables efficient and dynamic fusion tailored to the characteristics of local and global features. Unlike conventional fixed fusion strategies that lack input awareness, the Spatial-Channel Attention Fusion submodule within CAF adaptively adjusts spatial and channel-level weights, thereby preserving critical biological information and

avoiding indiscriminate feature merging. In addition to this adaptive fusion, we further introduce the Condition-Aware Dual Weight Optimization submodule, which further refines the fused representation by reweighting spatial and channel contributions based on global context. This two-stage fusion design enables DAISY to enhance feature synergy and maintain robust performance across diverse prediction scenarios. More importantly, DAISY provides intuitive and powerful interpretability, benefiting from its computer vision–inspired architecture, which inherently offers a distinct advantage in visualization. In contrast to existing models that typically rely on attention maps or mutation analysis for interpretability, DAISY can intuitively visualize TCR-epitope binding sites using the Score-CAM method[8], offering a high degree of transparency in its decision-making process.

In summary, DAISY integrates physicochemical properties into a biologically informed architecture, serving as a powerful tool for understanding TCR–epitope interactions and advancing personalized immunotherapy.

4. Utilization of CDR3β sequences and construction of the training TCR–Epitope Dataset

T cells play a fundamental role in adaptive immunity, primarily through antigen recognition mediated by αβ T-cell receptors (TCRs). Each TCR consists of α and β chains, with three complementarity-determining regions (CDRs) in each[9] (Figure S1). Among them, the CDR3β loop is the most variable and is widely recognized as the key determinant of peptide–MHC recognition due to its central role in contacting antigenic peptides. Based on this biological rationale, we utilized only the CDR3β sequence to

represent TCRs in our model, focusing on its potential to capture antigen specificity[10].

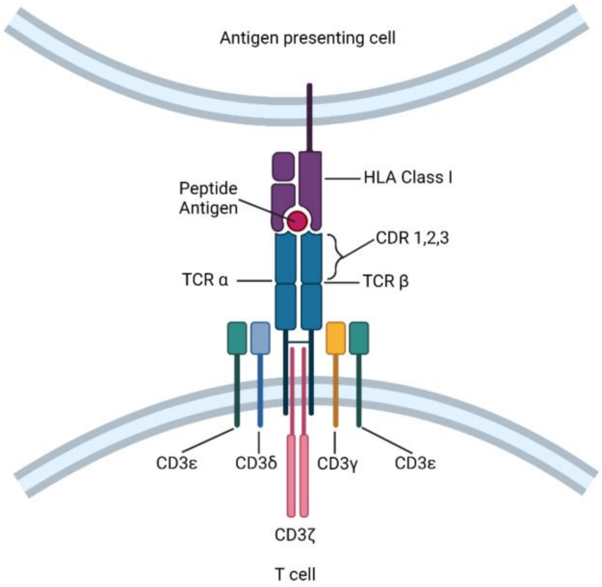

Figure S1. The T cell receptor (TCR) structure. The TCR comprises an α chain and a β chain.

To construct the TCR–epitope interaction dataset, we aggregated data from four publicly available databases (see Table S1). From McPAS-TCR[11] and PIRD[12], we collected 39,985 and 51,139 TCR–epitope pairs, respectively. An additional 197,229 pairs were retrieved from the IEDB database[13] using the following filters: "Linear Epitope," "Has Receptor Sequence," "Receptor Type: TCR αβ," "Positive Assays Only," "T Cell Assays," "MHC Class I," "Humans," and no restriction on disease or reference type. From VDJdb[14], 71,799 TCR–epitope pairs were extracted by filtering for human species, TRB gene usage, and MHC class I restriction. After merging the four datasets and removing duplicates, we obtained a final set of 70,469 unique positive TCR–epitope pairs, consisting of 64,166 unique CDR3β sequences and 127 unique epitopes.

5. Data exploration of the TCR-epitope data sets

To characterize the underlying structure of our curated datasets, we examined the sequence length distributions of CDR3β loops and epitope peptides across both the training set (Tr-TCR–epitope) and four independent test sets (Seen-Pair, Unseen-TCR, Unseen-Epitope, and Unseen-Pair). As shown in Figure S2, the CDR3β sequences

exhibit a relatively broad length distribution, with most sequences ranging from 12 to 18 amino acids. This variation reflects the natural diversity of TCR repertoires, particularly within the hypervariable CDR3 region. Conversely, epitope peptides display a highly concentrated distribution, predominantly centered at 9 and 10 amino acids, consistent with the canonical binding preferences of MHC class I molecules.

Table S1. Number of positive TCR–epitope pairs in each dataset used in this study.

Dataset	Positive
Tr-TCR-epitope	70,469
Seen-Pair	509
Unseen-TCR	589
Unseen-Epitope	434
Unseen-Pair	399

These observed patterns underscore two important biological properties of TCR–pMHC interactions: (i) the sequence variability of TCR CDR3β regions, which supports a wide antigen recognition spectrum, and (ii) the structural constraints imposed by the MHC binding groove on peptide length. Together, this analysis supports our modeling approach and informs data preprocessing strategies such as sequence normalization, input encoding, and model regularization.

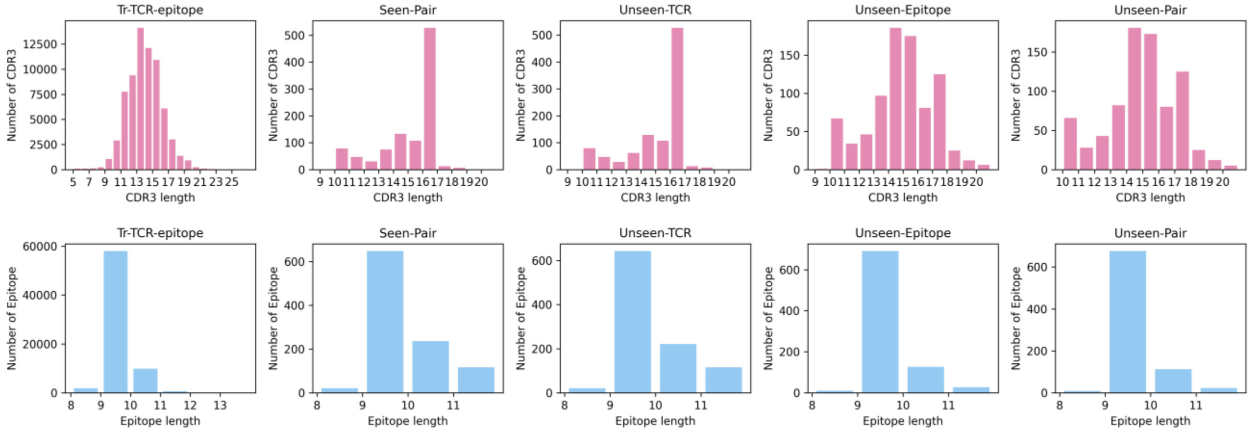

Figure S2. Length distributions of CDR3β and epitope in the TCR–epitope datasets. The left column shows the distributions of CDR3β lengths, and the right

column shows those of epitope lengths, across five datasets: the training set (Tr-TCR-pMHC) and four independent test sets (Seen-Pair, Unseen-TCR, Unseen-Epitope, Unseen-Pair).

6. Data exploration of the TCR-epitope data sets

To rigorously assess model generalization beyond the training domain, we evaluated all methods on four independent datasets: Seen-Pair, Unseen-TCR, Unseen-Epitope, and Unseen-Pair. As summarized in Figure S3, DAISY consistently outperformed all competing approaches, demonstrating both stability and robustness across diverse evaluation scenarios. On the Seen-Pair dataset, DAISY attained the highest performance with a ROC-AUC of 0.945 and PR-AUC of 0.954, substantially surpassing TITAN-PT[15] (ROC-AUC: 0.833, PR-AUC: 0.760) and TEIM[16] (ROC-AUC: 0.803, PR-AUC: 0.824), thereby underscoring its superior predictive capability. On the Unseen-TCR dataset, where most baselines exhibited moderate performance degradation relative to the Seen-Pair benchmark, DAISY maintained a marked advantage with a ROC-AUC of 0.945 and PR-AUC of 0.958. The strongest baseline, TEIM, achieved only 0.826 in ROC-AUC and 0.856 in PR-AUC, while TEIM and pMTnet[17] displayed further declines, highlighting the limited transferability of pretrained or specialized models. The Unseen-Epitope dataset posed a more stringent challenge, leading to pronounced performance reductions across nearly all baselines. DAISY, however, continued to dominate with the highest ROC-AUC (0.952) and PR-AUC (0.955). By contrast, TITAN-PT, the next best-performing method, reached significantly lower scores of 0.831 (ROC-AUC) and 0.742 (PR-AUC). Other models, including TEINet[18] and pMTnet, fell below a ROC-AUC of 0.68, indicating limited generalization capacity to novel epitope contexts. Finally, evaluation on the most challenging Unseen-Pair dataset further demonstrated DAISY's robustness. DAISY achieved a ROC-AUC of 0.965 and PR-AUC of 0.974, maintaining a substantial margin over all competitors. The strongest baseline, TITAN-PT, obtained a ROC-AUC of 0.826 and PR-AUC of 0.739, whereas TEIM and pMTnet yielded considerably lower values (0.810/0.839 and

0.721/0.629, respectively). The remaining methods performed near random classification, underscoring the pronounced performance gap. Collectively, these results demonstrate that DAISY achieves consistently superior generalization across heterogeneous evaluation scenarios, setting a new benchmark for reliable TCR–epitope prediction.

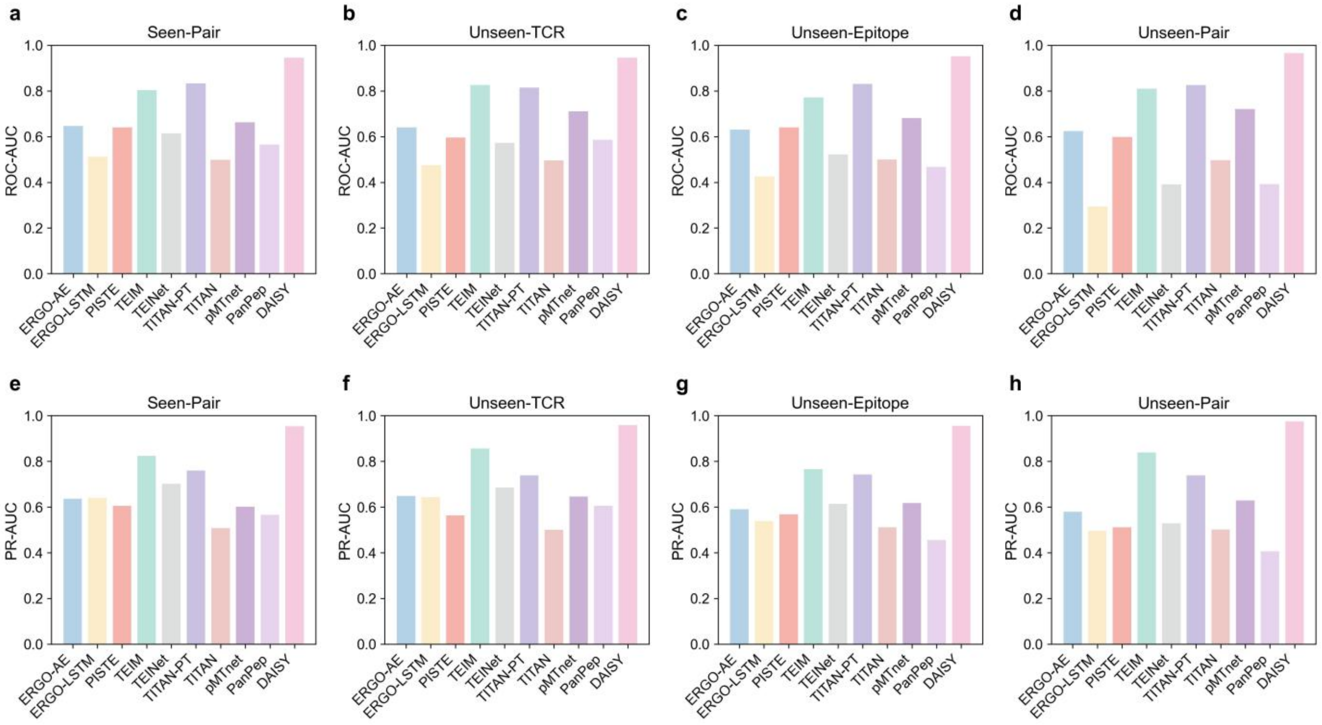

Figure S3. The performance comparison of DAISY and nine baseline models across four independent test sets. a, e: ROC-AUC and PR-AUC on the Seen-Pair test set; b, f: ROC-AUC and PR-AUC on the Unseen-TCR test set; c, g: ROC-AUC and PR-AUC on the Unseen-Epitope test set; d, h: ROC-AUC and PR-AUC on the Unseen-Pair test set. DAISY consistently outperforms all baselines across all evaluation scenarios, especially in settings involving completely novel TCRs or epitopes, demonstrating superior generalizability and robustness.

7. Score-CAM-Based Visualization of Model Attention

Score-CAM (Score-weighted Class Activation Mapping) was used to visualize the attention regions of the DAISY model[8]. As a gradient-free, activation-based method, Score-CAM computes class-specific heatmaps by linearly weighting the activation

maps from a convolutional layer based on the change in output score when masking the input with each activation. In this study, Score-CAM was applied to the final convolutional feature maps of the trained DAISY model, which takes TCR–epitope interaction maps as input. For each input pair, the model's output score was used to compute the importance of each spatial location, producing a heatmap that reflects residue-level attention. The heatmaps were then upsampled to the resolution of the original interaction maps for visualization.

A dataset of 112 TCR–epitope pairs with available 3D structures was obtained from STCRdab[19] and the Protein Data Bank (PDB). PyMOL was used to visualize and align the structures for comparative analysis.

8. In Silico Alanine Scanning Mutagenesis

In silico alanine scanning was performed on the same 112 TCR–epitope pairs. Residues in the CDR3β region of the TCR and the epitope sequence were selected for mutation if their corresponding Score-CAM activation values exceeded 0.56. For each selected residue, a single-point alanine mutation was simulated by modifying the input interaction map[20]. The mutated interaction maps were then passed through the trained DAISY model to obtain updated prediction scores. Score differences between wild-type and mutant inputs were recorded for all samples.

9. SHAP-based feature attribution analysis on negative samples

To explore DAISY’s attribution behavior on non-binding interactions, we conducted SHAP analysis[21] on 200 randomly selected negative samples. As shown in Figure S4a, the aliphatic index emerged as the most influential feature compared to positive samples, followed by molecular weight and instability index. Notably, features such as autocorrelation and hydrophobic moment exhibited near-zero attribution, suggesting limited relevance for predicting non-binding interactions. In Figure S4b, local properties such as mean fractional area loss and hydrophobicity remained influential, while flexibility exhibited increased attribution relative to positive samples. Overall, the divergence in feature importance highlights DAISY’s ability to adaptively leverage

distinct physicochemical properties under varying binding contexts.

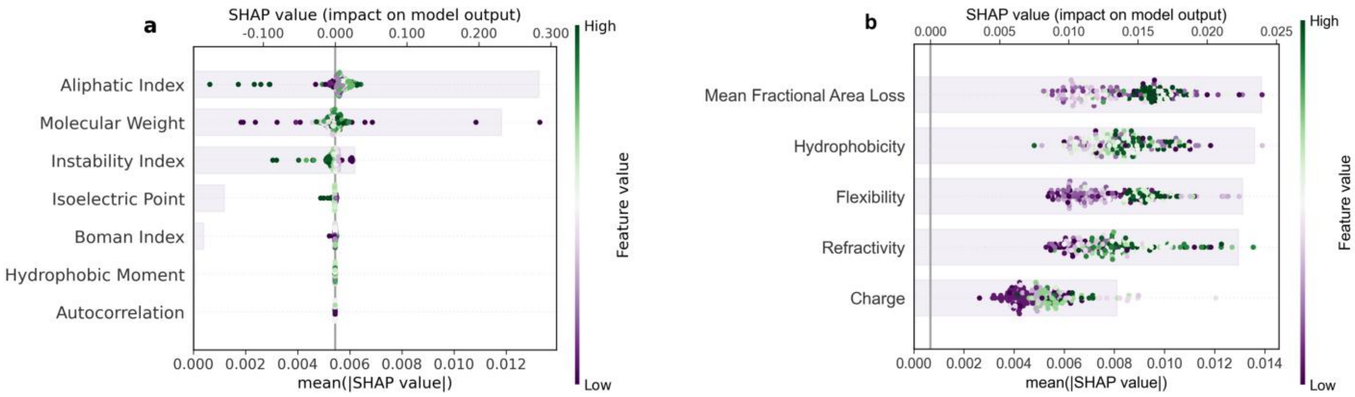

Figure S4. SHAP analysis on negative TCR-epitope pairs. a, SHAP summary plot of global physicochemical properties on negative TCR-epitope pairs. b, SHAP summary plot of local physicochemical properties on negative TCR-epitope pairs. Each dot represents the SHAP value of a single sample for the corresponding feature, with color indicating the feature value (from low to high, colored from purple to green). The accompanying bar chart displays the mean absolute SHAP value for each feature, reflecting its overall contribution to the model's prediction.

10. Feature ablation results on the Seen-Pair and Unseen-TCR sets

We also evaluated the effects of feature ablation on the Seen-Pair and Unseen-TCR datasets. Interestingly, removing hydrophobic moment led to comparable or marginally improved performance across several metrics in both datasets (Figure S5). This observation suggests that under low-challenge conditions, certain features may introduce minor redundancy or even contribute to overfitting. In contrast, removing autocorrelation resulted in a consistent performance drop even in these relatively stable settings, underscoring its broad contextual importance. Taken together, these findings highlight that hydrophobic moment is selectively beneficial, primarily under challenging scenarios, whereas autocorrelation remains broadly essential across both familiar and novel prediction settings.

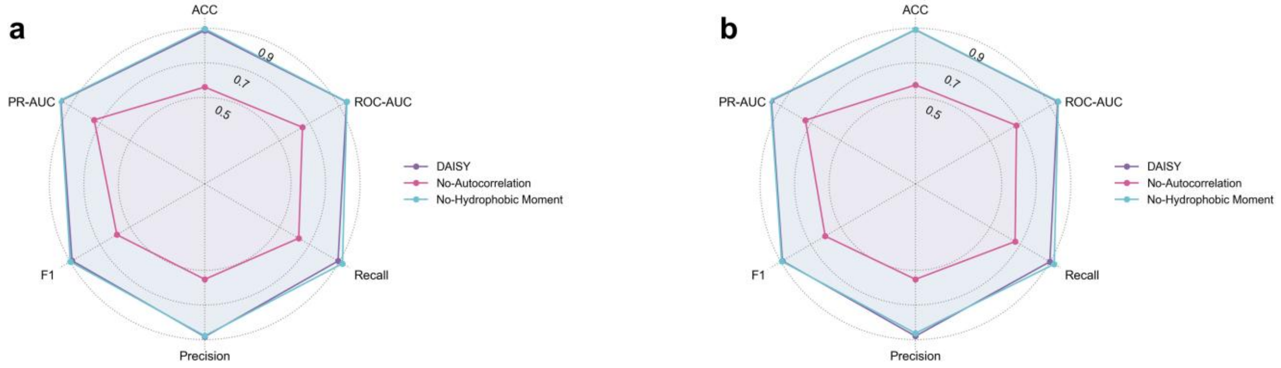

Figure S5. Feature ablation results across various evaluation settings. a, Radar plot comparing the performance of DAISY with its ablated versions on the Seen-Pair dataset. b, Radar plot comparing the performance of DAISY with its ablated versions on the Unseen-TCR dataset. No-Autocorrelation: model without autocorrelation; No-Hydrophobic Moment: model without hydrophobic moment. Metrics include accuracy (ACC), ROC-AUC, PR-AUC, precision, recall, and F1-score.

11. Data curation of the 10x Genomics Chromium cohort

We curated single-cell immune profiling data from the 10x Genomics Chromium Single Cell Immune Profiling platform (https://www.10xgenomics.com/products/single-cell-immune-profiling), which generates paired 5′ gene expression and V(D)J-enriched libraries for TCR sequencing using feature barcode technology. In this cohort, $CD8^{+}$ T cells from four healthy donors were profiled against 44 pMHC complexes derived from cancer and viral antigens (e.g., CMV, EBV, influenza, HTLV, HPV, HIV) using dCODE™ Dextramer reagents. T-cell binding specificity to each pMHC was quantified by Unique Molecular Identifier (UMI) counts. Previous studies applied stringent thresholds (UMI $\geqslant$ 10 and $\geqslant$ 5-fold higher than the maximum negative control), which may have excluded weaker but biologically relevant interactions. To minimize potential false negatives, we adopted a relaxed threshold (UMI >1) to retain both strong and weak binding events. The curated dataset

was then used to assess whether binding scores predicted by DAISY correlate with T-cell clonal expansion, by comparing clonotype fractions with predicted scores and calculating Spearman correlation coefficients with associated two-sided P values.

12. Clinical Outcome Prediction Analysis

✧ **Clinical Cohort and Data Processing:**

We utilized a publicly available dataset of 29 advanced melanoma patients treated with anti-PD-1 therapy, originally published by Riaz et al[22]. Whole-exome and RNA sequencing data, including TCR repertoires and clinical response information (CR/PR/SD/PD) and overall survival, were downloaded from https://github.com/riazn/bms038_analysis.

✧ **Definition of Functional and Conventional Biomarkers:**

Conventional Biomarkers: Tumor Mutational Load (TML) was defined as the total number of non-synonymous somatic mutations. Neoantigen Load (NAL) was the total count of predicted neoantigens[23–26].

✧ **DAISY-derived Functional Biomarkers:**

Immunogenic Neoantigen Load (INAL): For each patient, INAL was defined as the number of unique neoantigens predicted by DAISY to be recognized by at least one high-frequency T-cell clonotype. A TCR-neoantigen pair was considered recognized if the DAISY score exceeded a stringent threshold (e.g., > 0.9) to ensure high confidence. High-frequency clonotypes were defined as those constituting the top [e.g., 2000] of the productive T-cell repertoire by abundance.

Responsive T-cell Clonotype Frequency (RTCF): RTCF was calculated as the cumulative frequency of all T-cell clonotypes that were predicted by DAISY to be reactive to at least one neoantigen identified in the patient's tumor.

✧ **Statistical Analysis:**

Biomarker levels between responder (CR/PR) and non-responder (SD/PD) groups were compared using a one-sided Wilcoxon rank-sum test. For survival analysis, patients were stratified into 'High' and 'Low' groups based on the median value of each biomarker. Overall survival curves were generated using the Kaplan-

Meier method and compared with the log-rank test. Univariate Cox proportional hazards models were used to calculate Hazard Ratios (HRs).

Supplementary Tables

Table S2. Performance comparison of DAISY and its ablated variants under the Seen-Pair setting, where the GFA, SCAF, DANet, and CADO modules are either removed or incrementally added. The best scores are highlighted in bold.

Model	ACC	AUC	F1	AUPRC
ResNet-18	0.776	0.906	0.795	0.936
ResNet-GFA	0.825	0.940	0.838	0.957
ResNet-GFA-SCAF	0.832	0.936	0.844	0.959
ResNet-GFA-DANet	0.704	0.921	0.760	0.947
ResNet-GFA-CADO	0.697	0.874	0.750	0.903
ResNet-GFA-SCAF-DANet	0.739	0.917	0.783	0.941
DAISY	**0.887**	**0.949**	**0.888**	**0.962**

Table S3. Performance comparison of DAISY and its ablated variants under the Unseen-TCR setting, where the GFA, SCAF, DANet, and CADO modules are either removed or incrementally added. The best scores are highlighted in bold.

Model	ACC	AUC	F1	AUPRC
ResNet-18	0.782	0.918	0.799	0.941
ResNet-GFA	0.828	0.947	0.839	0.961
ResNet-GFA-SCAF	0.834	0.940	0.844	0.961
ResNet-GFA-DANet	0.699	0.923	0.754	0.947
ResNet-GFA-CADO	0.697	0.875	0.747	0.902
ResNet-GFA-SCAF-DANet	0.746	0.922	0.785	0.942
DAISY	**0.891**	**0.952**	**0.890**	**0.964**

Table S4. Performance comparison of DAISY and its ablated variants under the Unseen-Epitope setting, where the GFA, SCAF, DANet, and CADO modules are either removed or incrementally added. The best scores are highlighted in bold.

Model	ACC	AUC	F1	AUPRC
ResNet-18	0.804	0.858	0.804	0.896
ResNet-GFA	0.822	0.882	0.821	0.914
ResNet-GFA-SCAF	0.826	0.909	0.831	0.937
ResNet-GFA-DANet	0.752	0.871	0.777	0.910
ResNet-GFA-CADO	0.804	0.888	0.809	0.918
ResNet-GFA-SCAF-DANet	0.774	0.883	0.797	0.916
DAISY	**0.868**	**0.923**	**0.863**	**0.942**

Table S5. Local physicochemical properties (residue-level)

Name	Category	Biological Meaning
Hydrophobicity	Chemical	Reflects the tendency of an amino acid to avoid water; critical for protein folding and interface burial
Refractivity	Electronic/Structural	Related to molecular polarizability and side chain volume; important in shape complementarity
Charge	Electrostatic	Indicates the residue's electric charge at physiological pH; essential for ionic interactions
Flexibility	Structural	Estimates local backbone mobility; relevant for binding adaptability and loop movement
Mean Fractional Area Loss	Structural/Functional	Approximates loss of solvent-accessible surface area upon binding; reflects binding site burial

Table S6. Global physicochemical properties (sequence-level)

Name	Category	Biological Meaning
Isoelectric Point (pI)	Electrostatic	The pH at which the peptide carries no net charge; affects solubility and charge-mediated recognition
Molecular Weight	Structural	Sum of amino acid masses; influences molecular kinetics and stability
Instability Index	Structural	Predicts in vitro stability; values <40 suggest stable proteins
Aliphatic Index	Structural	Ratio of aliphatic side chains; correlates with thermal and structural stability
Boman Index	Functional	Estimates protein-binding potential; higher values imply stronger interaction capabilities
Hydrophobic Moment	Structural/Functional	Measures amphipathic character (esp. in helices); key for membrane/protein interface binding
Autocorrelation	Statistical/Functional	Captures spatial correlation of amino acid properties along the sequence; encodes periodicity/motifs

Table S7. Results of the Schoenfeld residual test for the proportional hazards assumption.

Biomarker	Schoenfeld P-value
TML	0.905
NAL	0.905
RTCF	0.932
INAL	0.962

References

1. Moris, P. *et al.* Current challenges for unseen-epitope TCR interaction prediction and a new perspective derived from image classification. *Briefings in Bioinformatics* **22**, (2021).
2. Dash, P. *et al.* Quantifiable predictive features define epitope-specific T cell receptor repertoires. *Nature* **547**, 89–93 (2017).
3. Rudolph, M. G., Stanfield, R. L. & Wilson, I. A. HOW TCRS BIND MHCS, PEPTIDES, AND CORECEPTORS. *Annual Review of Immunology* **24**, 419–466 (2006).
4. CD28 Costimulation: From Mechanism to Therapy. *Immunity* **44**, 973–988 (2016).
5. Zhu, J. & Paul, W. E. Heterogeneity and plasticity of T helper cells. *Cell Res* **20**, 4–12 (2010).
6. He, K., Zhang, X., Ren, S. & Sun, J. Deep Residual Learning for Image Recognition. Preprint at https://doi.org/10.48550/arXiv.1512.03385 (2015).
7. Vaswani, A. *et al.* Attention is all you need. in *Proceedings of the 31st International Conference on Neural Information Processing Systems* 6000–6010 (Curran Associates Inc., Red Hook, NY, USA, 2017).
8. Wang, H. *et al.* Score-CAM: Score-Weighted Visual Explanations for Convolutional Neural Networks. Preprint at https://doi.org/10.48550/arXiv.1910.01279 (2020).
9. Waldman, A. D., Fritz, J. M. & Lenardo, M. J. A guide to cancer immunotherapy: from T cell basic science to clinical practice. *Nat Rev Immunol* **20**, 651–668 (2020).
10. Sun, Y. *et al.* Evolution of CD8+ T Cell Receptor (TCR) Engineered Therapies for the Treatment of Cancer. *Cells* **10**, 2379 (2021).

11. Tickotsky, N., Sagiv, T., Prilusky, J., Shifrut, E. & Friedman, N. McPAS-TCR: a manually curated catalogue of pathology-associated T cell receptor sequences. *Bioinformatics* **33**, 2924–2929 (2017).

12. Zhang, W. *et al.* PIRD: Pan Immune Repertoire Database. *Bioinformatics* **36**, 897–903 (2020).

13. Vita, R. *et al.* The Immune Epitope Database (IEDB): 2018 update. *Nucleic Acids Research* **47**, D339–D343 (2019).

14. Bagaev, D. V. *et al.* VDJdb in 2019: database extension, new analysis infrastructure and a T-cell receptor motif compendium. *Nucleic Acids Research* **48**, D1057–D1062 (2020).

15. Weber, A., Born, J. & Rodriguez Martínez, M. TITAN: T-cell receptor specificity prediction with bimodal attention networks. *Bioinformatics* **37**, i237–i244 (2021).

16. Peng, X. *et al.* Characterizing the interaction conformation between T-cell receptors and epitopes with deep learning. *Nat Mach Intell* **5**, 395–407 (2023).

17. Lu, T. *et al.* Deep learning-based prediction of the T cell receptor–antigen binding specificity. *Nat Mach Intell* **3**, 864–875 (2021).

18. Jiang, Y., Huo, M. & Cheng Li, S. TEINet: a deep learning framework for prediction of TCR–epitope binding specificity. *Briefings in Bioinformatics* **24**, bbad086 (2023).

19. Leem, J., de Oliveira, S. H. P., Krawczyk, K. & Deane, C. M. STCRDab: the structural T-cell receptor database. *Nucleic Acids Res* **46**, D406–D412 (2018).

20. Weiss, G. A., Watanabe, C. K., Zhong, A., Goddard, A. & Sidhu, S. S. Rapid mapping of protein functional epitopes by combinatorial alanine scanning.

Proceedings of the National Academy of Sciences **97**, 8950–8954 (2000).

21. Lundberg, S. & Lee, S.-I. A Unified Approach to Interpreting Model Predictions. Preprint at https://doi.org/10.48550/arXiv.1705.07874 (2017).

22. Tumor and Microenvironment Evolution during Immunotherapy with Nivolumab. *Cell* **171**, 934-949.e16 (2017).

23. Zou, X. *et al.* Prognostic Value of Neoantigen Load in Immune Checkpoint Inhibitor Therapy for Cancer. *Front. Immunol.* **12**, (2021).

24. Garsed, D. W. *et al.* The genomic and immune landscape of long-term survivors of high-grade serous ovarian cancer. *Nat Genet* **54**, 1853–1864 (2022).

25. Samstein, R. M. *et al.* Tumor mutational load predicts survival after immunotherapy across multiple cancer types. *Nat Genet* **51**, 202–206 (2019).

26. Dall'Olio, F. G. *et al.* Tumour burden and efficacy of immune-checkpoint inhibitors. *Nat Rev Clin Oncol* **19**, 75–90 (2022).
